# Molecular and *in vivo* phenotyping of missense variants of the human glucagon receptor

**DOI:** 10.1016/j.jbc.2021.101413

**Published:** 2021-11-19

**Authors:** Wijnand J.C. van der Velden, Peter Lindquist, Jakob S. Madsen, Roderick H.M.J. Stassen, Nicolai J. Wewer Albrechtsen, Jens J. Holst, Alexander S. Hauser, Mette M. Rosenkilde

**Affiliations:** 1 Laboratory for Molecular Pharmacology, Department of Biomedical Sciences, Faculty of Health and Medical Sciences, University of Copenhagen, Copenhagen, Denmark; 2 Department of Drug Design and Pharmacology, Faculty of Health and Medical Sciences, University of Copenhagen, Copenhagen, Denmark; 3 Department of Biomedical Sciences, Faculty of Health and Medical Sciences, University of Copenhagen, Copenhagen, Denmark; 4 Novo Nordisk Foundation Center for Protein Research, Faculty of Health and Medical Sciences, University of Copenhagen, Copenhagen, Denmark; 5 Department of Clinical Biochemistry, Rigshospitalet, University of Copenhagen, Copenhagen, Denmark; 6 Novo Nordisk Foundation Center for Basic Metabolic Research, Faculty of Health and Medical Sciences, University of Copenhagen, Copenhagen, Denmark

**Keywords:** glucagon receptor, GCGR, single nucleotide polymorphism, missense variants, Gɑs signaling, glucagon binding, β-arrestin, class B1, GPCR, pharmacogenomics, AUC, area under the curve, BMI, body mass index, CI, confidence interval, DMEM, Dulbecco's modified Eagle's medium, ECD, extracellular domain, ECL, extracellular loop, GIPR, GIP receptor, GLP-1, glucagon-like peptide-1, GLP-1R, glucagon-like peptide-1 receptor, gnomAD, genome aggregation database, GPCR, G protein–coupled receptor, LoF, loss of function, MAF, minor allele frequency, PEI, polyethylenimine, TM, transmembrane

## Abstract

Naturally occurring missense variants of G protein–coupled receptors with loss of function have been linked to metabolic disease in case studies and in animal experiments. The glucagon receptor, one such G protein–coupled receptor, is involved in maintaining blood glucose and amino acid homeostasis; however, loss-of-function mutations of this receptor have not been systematically characterized. Here, we observed fewer glucagon receptor missense variants than expected, as well as lower allele diversity and fewer variants with trait associations as compared with other class B1 receptors. We performed molecular pharmacological phenotyping of 38 missense variants located in the receptor extracellular domain, at the glucagon interface, or with previously suggested clinical implications. These variants were characterized in terms of cAMP accumulation to assess glucagon-induced Gα_s_ coupling, and of recruitment of β-arrestin-1/2. Fifteen variants were impaired in at least one of these downstream functions, with six variants affected in both cAMP accumulation and β-arrestin-1/2 recruitment. For the eight variants with decreased Gα_s_ signaling (D63^ECD^N, P86^ECD^S, V96^ECD^E, G125^ECD^C, R225^3.30^H, R308^5.40^W, V368^6.59^M, and R378^7.35^C) binding experiments revealed preserved glucagon affinity, although with significantly reduced binding capacity. Finally, using the UK Biobank, we found that variants with wildtype-like Gα_s_ signaling did not associate with metabolic phenotypes, whereas carriers of cAMP accumulation-impairing variants displayed a tendency toward increased risk of obesity and increased body mass and blood pressure. These observations are in line with the essential role of the glucagon system in metabolism and support that Gα_s_ is the main signaling pathway effecting the physiological roles of the glucagon receptor.

Glucagon is a 29–amino acid peptide hormone that is secreted by the α-cells of the pancreatic islets upon decreases in blood glucose. In the liver, glucagon promotes glucose production, whereas in a feedback loop, glucose inhibits glucagon secretion (1, 2). Therefore, antagonists of the glucagon receptor have been proposed as glucose-lowering drugs in diabetes (3). In another feedback cycle, glucagon promotes hepatic amino acid metabolism, whereas amino acids stimulate glucagon secretion (4, 5). Dysregulation of this system can lead to elevated plasma concentrations of amino acids (hyperaminoacidemia) because of impaired ureagenesis in the liver. The elevated amino acids in turn increase the secretion of glucagon causing hyperglucagonemia. Hyperaminoacidemia may also cause proliferation of pancreatic α-cells, thereby further aggravating the hyperglucagonemia. The hyperglucagonemia may result in hyperglycemia as indicated by the glucose-lowering effects of glucagon antagonists in diabetic hyperglycemia (2, 6, 7, 8).

The glucagon receptor is primarily expressed on hepatocytes and to a lesser extent in the pancreas, kidneys, adipose tissue, and heart (9). A recent study describes how glucagon, besides activating the glucagon receptor, also activates the glucagon-like peptide-1 (GLP-1) receptor (GLP-1R), for instance, in the pancreatic β-cells as part of an intraislet communication between the α- and β-cells (10). Both receptors belong to class B1 of the superfamily of G protein–coupled receptors (GPCRs). In contrast to class A GPCRs, the receptors belonging to this class possess a much larger N-terminus, constituting the extracellular domain (ECD). The ECD is essential for the initial ligand–receptor recognition in which the ligand’s C terminus interacts with the ECD, after which the N-terminus of the ligand docks into the transmembrane (TM) domain of the receptor to induce conformational changes resulting in receptor activation (11). Subsequently, the receptor couples to heterotrimeric G proteins to initiate signaling and downstream gene transcription (12). As for the other class B1 receptors, the glucagon receptor mainly binds G protein αs (Gα_s_), which leads to an activation of adenylate cyclase and subsequent increase in downstream cAMP levels (13). This in turn activates protein kinase A signaling, thereby inhibiting glycolysis, stimulating glucose production, and raising blood glucose levels (14). Besides Gα_s_, the glucagon receptor has also been reported to bind Gα_q/11_ and Gα_i/o_ (15, 16).

Another common phenomenon for class B1 receptors is their maintained but low potent β-arrestin recruitment as opposed to their high potency in G protein coupling (17, 18). β-Arrestins bind sterically to the receptor, thereby hampering the receptor–G protein interaction and the subsequent (agonist-mediated) G protein signaling. For some class B1 receptors, β-arrestins are needed for internalization, as seen with the GIP receptor (GIPR) (18, 19), whereas GLP-1R internalizes independently of β-arrestins (19, 20, 21). The role of arrestin recruitment for internalization of the glucagon receptor is currently not well established (22).

Recently, efforts have been made to characterize identified genetic missense variants of GPCRs and their cognate ligands (23, 24), as these may be associated with altered receptor function and selectivity, which in turn could contribute to disease and unintended drug responses (25). This highlights the need for characterization of the cellular effects of receptor missense variants with respect to ligand binding and receptor signaling. So far, only a few missense variants have been identified in the glucagon receptor gene (GCGR). This concerns both heterozygous and homozygous carriers of missense variants identified in cohort studies and single-case studies. Some of these variants have also been studied in genetically engineered animals. Taken together, G40^ECD^S, D63^ECD^N, P86^ECD^S, V368^6.59^M, and the double variant R225^3.30^H–V368^6.59^M (Wootten nomenclature in superscript (26)) have all been linked to metabolic disorders, with phenotypes comprising non-insulin-dependent diabetes, hyperglucagonemia, and α-cell hyperplasia, putatively due to an impaired receptor signaling and/or decreased binding capacity of glucagon (27, 28, 29, 30, 31, 32, 33).

In the present study, we describe the glucagon binding and receptor function of a series of missense variants from genome aggregation database (gnomAD), which comprises around 140,000 exome and whole-genome sequences across seven subpopulations and their association with cardiometabolic phenotypic traits (https://gnomad.broadinstitute.org/).

## Results

### Class B1 mutational constraint spectrum

**Figure 1:**
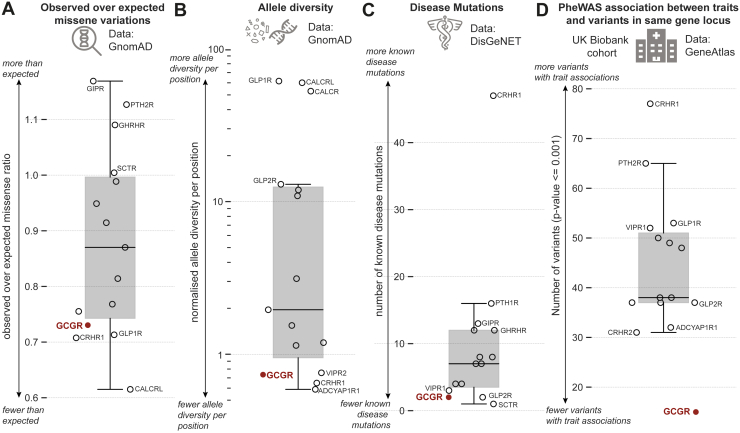
**Class B1 mutational constraint spectrum.***A*, observed over expected class B1 missense variants as observed in gnomAD (34). *B*, allele diversity per residue position in each receptor normalized by receptor length. *C*, known disease mutations for each receptor from DisGeNET (36). *D*, number of variants with trait associations as obtained from GeneAtlas (39). ADCYAP1R1, pituitary adenylate cyclase-activating polypeptide type I receptor; CALCR, calcitonin receptor; CALCRL, calcitonin receptor-like receptor; CRHR1, corticotropin-releasing hormone receptor 1; GCGR, glucagon receptor (highlighted); GHRHR, growth hormone releasing hormone receptor; GIPR, glucose-dependent insulinotropic polypeptide receptor; GLP-1R, glucagon-like peptide-1 receptor; GLP-2R, glucagon-like peptide-2 receptor; PTH2R, parathyroid hormone 2 receptor; SCTR, secretin receptor; VIPR1, vasoactive intestinal peptide receptor 1; VIPR2, vasoactive intestinal peptide receptor 2.

First, we obtained aggregated data of natural genetic variations and of missense variant's mutational constraint spectrum for all class B1 GPCRs from gnomAD (34). Comparing the distribution of the ratio of observed over expected number of missense variants showed that among the class B1 receptors, the glucagon receptor, the GLP-1R, the corticotropin-releasing hormone receptor 1 (CRHR1), and the calcitonin receptor-like receptor displayed fewer missense variants than expected (*i.e.*, from neutrally evolving background) and compared with, for instance, the GIPR (Fig. 1*A*). This suggests that these four receptors are under a stronger evolutionary selection against missense variations owing to purging of deleterious variants from human populations (negative selection). Although there can be fewer variant positions than expected, variant allele frequency distributions can still vary significantly between receptors. To account for this, we calculated the mean allele frequency per missense variant position normalized by receptor length (Fig. 1*B*). Again, the glucagon receptor and CRHR1 ranked among the lowest ranking receptors with the least mean allele diversity per variant position. Previous studies have shown that allele frequencies segregating at a putatively functional site are a good indicator for functional importance and tend to occur at a lower frequency than at synonymous sites (35).

Next, we investigated the spectrum of known disease mutations among the class B1 receptors (Fig. 1*C*). Although there are known disease mutations for all class B1 receptors, the number of mutations and the diversity of the associated phenotypic traits were wide ranging. For instance, CRHR1 has more than 45 disease mutations reported with a broad diversity of disease manifestations, including depressive disorders, Parkinson’s disease, irritable bowel syndrome, asthma, and fragile X syndrome (36). The glucagon receptor ranked among the lowest on the list with only one known disease-associated mutation, rs1801483/G40^ECD^S associating with diabetes, recorded in DisGeNET (36). A recent study showed that mutation-tolerant genes exemplified by olfactory receptors (*i.e.*, those with fewer disease mutations) tend to function in metabolism, whereas more mutation-intolerant genes tended to function in development and signal transduction pathways (37). These findings imply a higher fitness cost for mutations in developmental than in metabolic genes (38).

Finally, we investigated how associations between hundreds of traits from the UK Biobank associated with variants (PheWAS) in class B1 receptors (Fig. 1*D*) (39). This showed a similar trend as for the disease mutations, where CRHR1 had many variants associated with a clinical trait, whereas the glucagon receptor again displayed fewer variants with trait associations.

### Mutational landscape of glucagon receptor missense variants

**Figure 2:**
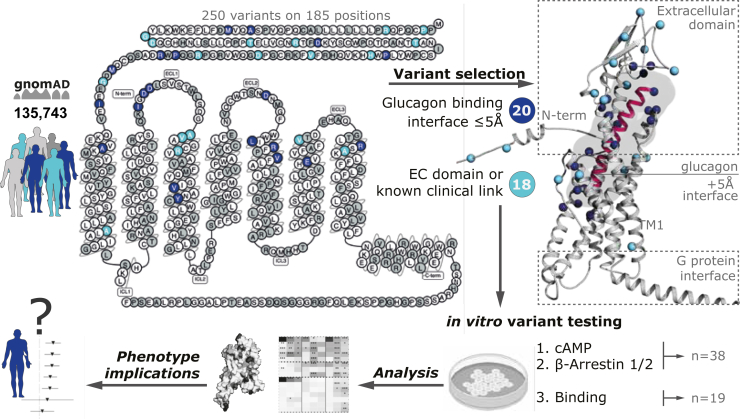
**Missense variants and selections from the human glucagon receptor.** All 250 glucagon receptor missense variants in 185 positions have been selected from the gnomAD cohort spanning 135,743 individuals or the literature depicted on a 2D snake-plot (34). We then selected variants in the binding interface (≤5 Å) of glucagon (n = 18, *dark blue*) by investigating all three glucagon-bound receptor structures (Protein Data Bank: 6LMK, 6LML, and 6WPW (16, 44)) and additional variants in the extracellular domain or from previous literature characterizations (n = 20, *light blue*). Structural mapping of the selected variants on the cryo-EM structure of the human glucagon receptor bound glucagon and Gα_s_ (6LMK) with a modeled AlphaFold2 disordered N terminus to illustrate two variants located outside the refined structure in the extracellular domain (84).

To further assess the mutational spectrum of the glucagon receptor, we identified all missense variants across the 135,743 individuals in gnomAD (https://gnomad.broadinstitute.org/). In addition, we included (P86^ECD^S), which has been reported in a clinical association study (28). Taken together, we identified 250 missense variants that were scattered across the receptor sequence with varying frequencies (Fig. 2 and Table S1).

Several studies of glucagon receptor structures have been published. This includes three structures in an inactive state (40, 41, 42); one that is thought to reflect an intermediate state, *i.e.*, the structure assumed upon binding of the glucagon analogue and partial agonist NNC1702 (43); and four structures in the fully activated state(s) after binding to the activating peptides, glucagon (Gα_s_ and Gα_i_), ZP3780 (a C-terminal modified glucagon), and the dual-agonist P15 (which also activates GLP-1R) (16, 44, 45). Based on examination of all three glucagon-bound receptor structures (Protein Data Bank accession number 6LMK, 6LML, and 6WPW (16, 44)) we selected 38 missense variants that were either (I) directly located within the glucagon-binding interface (≤5 Å) (n = 20), (II) in the ECD (n = 12), or (III) reported to have direct implications from receptor structure–function studies or clinical associations described in the literature (n = 6) (Fig. 2). All missense variants had an MAF (minor allele frequency) of <0.01, characterizing them as rare variants. Furthermore, distinctly few homozygous carriers were identified (0.60% of the total amount of carriers), which supports the observation that the glucagon receptor is under negative selection (Table S1).

Beyond the variants located in the binding interface or ECD, we included A220^3.25^V and V221^3.26^L as several studies on both the glucagon receptor and GLP-1R highlight these residues as important for receptor activation (40, 42, 46, 47). Similarly, A380^7.37^T was included as an essential contributor for activation of other class B1 receptors, like the secretin, GLP-1, and CRH receptors (48, 49, 50). The variant R225^3.30^H has been shown to contribute to the stabilization of receptor loop conformation important for ligand binding (40, 42). In addition, the double variant R225^3.30^H–V368^6.59^M has been clinically associated with α-cell hyperplasia in homozygous carrier(s) (29). V368^6.59^M, located at the beginning of extracellular loop (ECL) 3, was included as the most frequent in this region and is considered important for ligand and ECD recognition (51). Moreover, the corresponding variant in mice has been associated with hyperglucagonemia, α-cell hyperplasia, and pancreas enlargement (30). Finally, A159^1.57^T was selected due to the high conservation of an alanine at this position among class B1 receptors (52).

### Molecular phenotyping of selected missense variants

**Figure 3:**
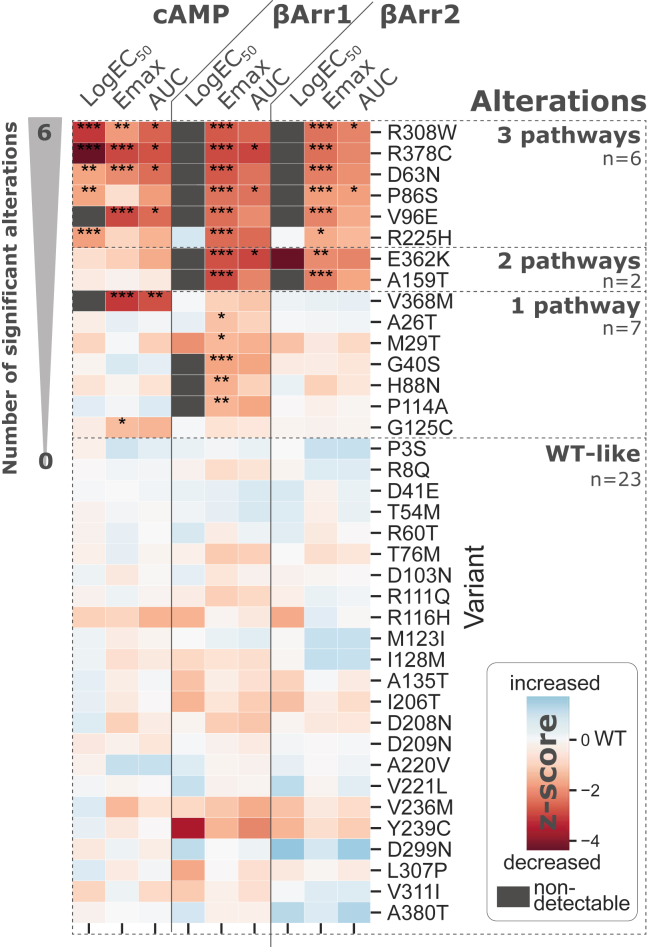
**Graphical overview of the molecular phenotyping of the 38 selected missense variants.** Area under the curve (AUC) of the dose–response curves, maximal response (E_max_), and LogEC_50_ between mutant and wildtype glucagon receptor in cAMP accumulation (wildtype n = 33; variants n = 3–6) and β-arrestin1/2 recruitment (wildtype n = 31; variants n = 3–5). Variants are sorted by their number of statistically significant (ordinary one-way ANOVA *p* < 0.05) alterations and clustered into four groups based on displaying wildtype-like signaling and their number of cross-pathway significant alterations. In total, 15 variants display altered signaling in at least one pathway measured endpoint. Individual parameters are Z-score normalized to compare between endpoints. AUC and Emax of variants were normalized to the wildtype glucagon receptor. ND (*gray background*) indicates nondetectable potency windows at 10 nM glucagon (for cAMP accumulation) and 10 μM glucagon (for the β-arrestin recruitment). ∗*p* < 0.05; ∗∗*p* < 0.005; ∗∗∗*p* < 0.0005.

The 38 selected missense variants were introduced in the wildtype human glucagon receptor, and cAMP accumulation measurements were performed to assess the impact of the mutations on Gα_s_-mediated signaling. We used transiently transfected COS-7 cells to determine glucagon’s potency (EC_50_), maximal receptor signal (E_max_), and the integrated response of these two parameters, *i.e.*, the area under the curve (AUC). The variants' ability to recruit β-arrestin-1 and -2 upon ligand-mediated receptor activation was tested in a bioluminescence resonance energy transfer–based assay using transiently transfected HEK293 cells. These functional experiments allowed us to cluster the variants depending on whether they are wildtype-like or significantly altered in one, two, or three pathways (Fig. 3 and Table S2). To compare variant effects across pathway measurements and pharmacological parameters, we normalized all measurements relative to wildtype presenting the result as a Z-score. This Z-score reflects the result expressed as the number of standard deviations above or below the wildtype value of that parameter.

Among the 38 selected missense variants, 23 variants were wildtype-like for all pathways, whereas 15 variants showed deviating activation profiles (Fig. 3). Seven variants were impaired in one pathway. This included G125^ECD^C and V368^6.59^M, which were impaired in cAMP accumulation, and A26^ECD^T, M29^ECD^T, G40^ECD^S, H88^ECD^N, P114^ECD^A, which had decreased β-arrestin-1 recruitment. Two variants, A159^1.57^T and E362^6.53^K were affected in both β-arrestin-1 and 2 recruitment, adding up to seven variants with a selective decrease in β-arrestin recruitment but wildtype-like cAMP responses. Lastly, six variants were altered in all three pathways: D63^ECD^N, P86^ECD^S, V96^ECD^E, R225^3.30^H, R308^5.40^C, and R378^7.35^C. Of interest, no variant exclusively affected Gα_s_ signaling and β-arrestin-2 recruitment without showing a concomitant effect on β-arrestin-1 recruitment. Finally, no variants showed enhanced responses in Gα_s_ signaling and/or β-arrestin1/2 recruitment.

### Signaling profiles for glucagon missense variants with distinct Gα_s_ activation

**Figure 4:**
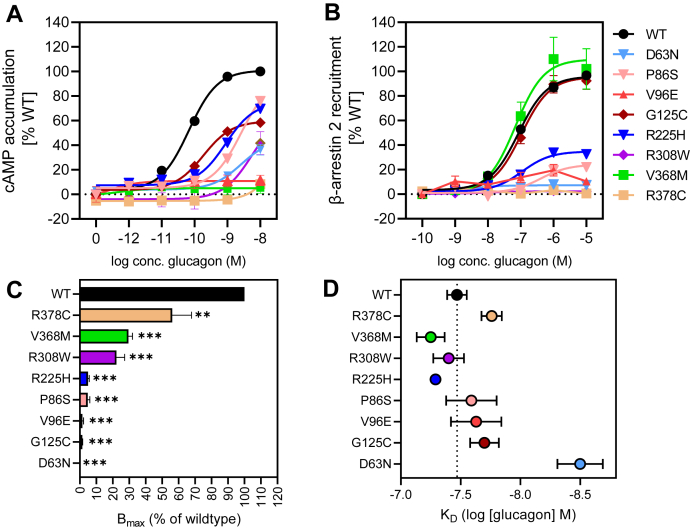
**Signaling and binding profiles of missense variants with significantly altered Gɑ**_**s**_**activation.** The eight missense variants (D63^ECD^N, P86^ECD^S, V96^ECD^E, G125^ECD^C, R225^3.30^H, R308^5.40^W, V368^6.59^M, and R378^7.35^C) displayed significantly (*p* < 0.05) decreased Gα_s_ activation. *A*, dose–response curves in cAMP accumulation for wildtype glucagon receptor and variants (wildtype n = 33, variants n = 3–6.). *B*, dose–response curves in β-arrestin-2 recruitment for wildtype glucagon receptor and variants (wildtype n = 31, variants n = 3–5). *C*, B_max_ values from homologous competition binding with [^125^I]glucagon and unlabeled glucagon for wildtype and receptor variants (wildtype n = 13; variants n = 3–5). *D*, the corresponding K_D_ values of the competition binding. Data represent the mean ± SEM of n independent experiments performed in duplicate.

Given that Gα_s_ activation within the cell is linked to physiological processes (14), we explored more closely the signaling profiles for the eight missense variants that had lower Gα_s_ activation (D63^ECD^N, P86^ECD^S, V96^ECD^E, G125^ECD^C, R225^3.30^H, R308^5.40^W, V368^6.59^M, and R378^7.35^C; Fig. 4*A* and Table S2). Among these are V96^ECD^E and V368^6.59^M, which had nondetectable Gα_s_ activation at 10 nM glucagon. Previously, Lin *et al.* (30) also reported decreased Gα_s_ activity for V368^6.59^M but less pronounced than observed here. To explore whether the loss of activity of V368^6.59^M was related to the transfection efficiency, we doubled the amount of DNA (20 μg) during transfection. Now, we observed a weak response at 10 nM for V368^6.59^M. This was comparable with the wildtype response with 5 μg of receptor DNA, suggesting V368^6.59^M’s signaling is indeed impaired, as much more receptor is required to reach a fraction of the wildtype activity (around 20%) (Fig. S1). Continuing our investigation, two other variants with highly impaired Gα_s_ response (R308^5.40^W and R378^7.35^C) exhibited a >1000-fold decrease in potency with a concomitant low activity at 10 nM glucagon (Fig. 4*A* and Table S2). For D63^ECD^N the effect on potency was less severe than for the previous two with a 219-fold shift in potency and a maximal effect of only 36% of wildtype. In the case of P86^ECD^S and R225^3.30^H, the decrease in potency was 19- and 86-fold, respectively, with a less pronounced impairment of maximal efficacy (statistically nonsignificant). G125^ECD^C was the only variant with maintained potency but a decrease in maximal activity amounting to around 58% of that of the wildtype.

Given the significantly altered Gα_s_ signaling for these eight missense variants, we extended the *in vitro* characterization and evaluated their ability to recruit β-arrestin-1 and -2 (Fig. 4*B* and Table S2). Six of eight variants (D63^ECD^N, P86^ECD^S, V96^ECD^E, R225^3.30^H, R308^5.40^W, and R378^7.35^C) showed significantly decreased maximal β-arrestin-2 recruitment, ranging from 0.6% to 32.8% of wildtype. For five of six variants, EC_50_ could not be determined, whereas for R225^3.30^H a 6-fold decrease was observed. More interestingly, G125^ECD^C and V368^6.59^M, both of which were impaired with respect to cAMP accumulation, acted similarly to wildtype in β-arrestin-2 recruitment, which may suggest coupling preferences for arrestin over Gα_s_.

To determine whether the impaired Gα_s_ activation of these eight variants was caused by a loss of glucagon binding, we determined glucagon’s affinity in a homologous competition binding assay. Here, all eight variants were able to bind glucagon (Fig. 4*C* and Table S3), however with lower maximal binding capacity (B_max_) as compared with wildtype. R308^5.40^W, V368^6.59^M and R378^7.35^C were the only variants with a B_max_ for glucagon higher than 20%. In terms of the binding affinity of glucagon for these genetic variants, seven of eight were able to bind glucagon with wildtype-like affinities for glucagon, except for D63^ECD^N (with a 10-fold increase) (Fig. 4*D* and Table S3). Noticeable, for this variant, the B_max_ was 0.2% of wildtype, suggesting that the altered affinity might be due to an extremely low B_max_. Altogether, genetic variants of the glucagon receptor may impair G protein activation through altered potency of glucagon, E_max_, or a combination hereof. Impairment in Gα_s_ signaling did not necessarily lead to decreased β-arrestin recruitment as seen for G125^ECD^C and V368^6.59^M. Lastly, receptor binding affinity was maintained for these genetic variants, even though Gα_s_ signaling was impaired. However, in all cases a lower B_max_ was observed.

### Structural fundament for the signaling phenotypes of the variants

**Figure 5:**
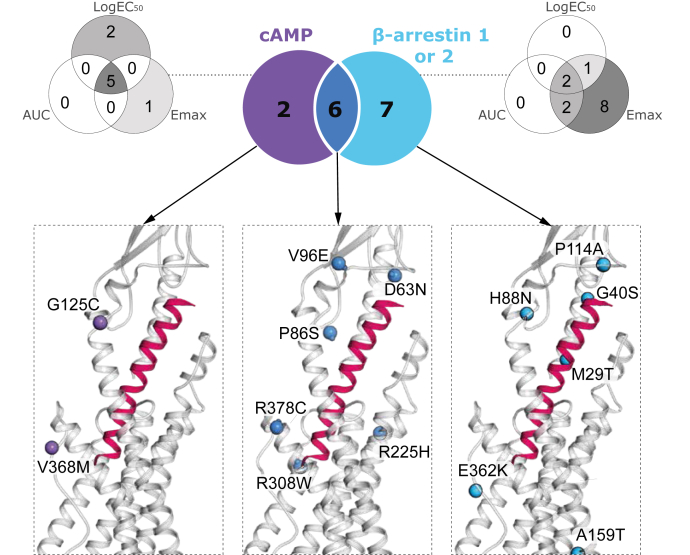
**Overlap between glucagon receptor missense variants and their effect on cAMP accumulation and beta-arrestin1/2 signaling.** Glucagon missense variants significantly affecting cAMP accumulation (*purple*), β-arrestin1/2 recruitment (*cyan*), or both pathways (*dark blue*). Missense variants found in different segments of the Venn diagram are visualized by 3D representation of glucagon in complex with the glucagon receptor and Gα_s_ protein (Protein Data Bank: 6LMK). Sub-Venn diagram for each pathway displays how individual missense variants affect one, two, or three pharmacological parameters (E_max_, EC_50_, and AUC).

We next focused on the structural basis for the altered signaling of these variants. To do so, we first clustered each pathway into sub-Venn diagrams to illustrate the distribution of missense variants that have a pooled impact on one, two, or three pharmacological parameters (Fig. 5). We then employed the cryo-EM structure of the glucagon receptor in complex with glucagon and Gα_s_ (Protein Data Bank: 6LMK) (16) to identify where the clusters of important residues are located. The missense variants in the N terminus (P3^ECD^S, R8^ECD^Q and A26^ECD^T) were not projected into this static structure, as these residue positions were not visualized in the cryo-EM structure.

For the two variants (G125^ECD^C and V368^6.59^M) with selectively impaired Gα_s_ (purple cluster; Fig. 5, left inset), G125^ECD^C (located in the transition to TM3) is proposed to be a part of the glucagon receptor stalk region (residue G125^ECD^–K136^1.34^) and thereby may be involved in the modulation of the active receptor conformation (42). The residue V368^6.59^ in TM6 has been shown to indirectly contribute to the stabilization and interaction of the N-terminus of glucagon and the ECL3 of the glucagon receptor by forming a hydrophobic pocket, which also consists of V364^6.55^, F367^6.58^, A373^ECL3^, L377^7.34^, and K381^7.38^ (30). The missense variant (V368^6.59^M) causes a disruption of this hydrophobic network, thereby increasing the distances between the ECL3 and the glucagon N-terminus but still allowing glucagon binding, highlighting the importance of ECL3 for Gα_s_ signaling.

Among the six missense variants affecting both cAMP accumulation and β-arrestin-1/2 recruitment (dark blue cluster; Fig. 5, middle inset), four variants (D63^ECD^N, P86^ECD^S, R308^5.40^W, R378^7.35^C) were located within the binding interface (≤5 Å), one variant (V96^ECD^E) was located in the ECD, and one variant (R225^3.30^H) was located in TM3. The corresponding residues potentially play a role in the stabilization of ECL1 and are therefore important for receptor activation. This was seen for R225^3.30^ since it makes a salt bridge with D218^ECL1^ in the crystal structure (40, 53). V96^ECD^ is located closely to R116^ECD^ and placed in the top part of the receptor, pointing toward the ligand with a distance of 2.4 Å to the established glucagon interaction residue M27^∗^. The corresponding missense variants V96^ECD^E and R116^ECD^H were highly impaired in Gα_s_ activation, suggesting that R116^ECD^ may contribute directly to the stabilization of this area by making direct contacts with the ligand, whereas V96^ECD^ interacts indirectly with the ligand. In addition, the impairment in signaling of R116^ECD^H agrees with other substitutions in the same position (40, 54). The homologous residue of P86^ECD^ in GIPR (P89^ECD^) and GLP-1R (P90^ECD^) was similarly found to be essential for the interaction with GIP and GLP-1, respectively, supporting a key role for this position in activation of class B1 receptors (55, 56).

Among the seven variants that exclusively impacted β-arrestin-1 and/or -2 recruitment (cyan cluster; Fig. 5, right inset), four (A26^ECD^T, M29^ECD^T, P114^ECD^A, and E362^6.53^K) were located within the binding interface (≤5 Å). Two variants (G40^ECD^S and H88^ECD^N) were in the ECD, whereas one (A159^1.57^T) was located in the lower part of TM1, where this position is conserved as an alanine among many class B1 GPCRs (52). Without having a direct interaction with the G protein, A159^1.57^T has been suggested to contribute indirectly to receptor activity through a hydrophobic network stabilizing an active conformation (16). Among the seven exclusively β-arrestin-affected variants, only two (A159^1.57^T and E362^6.53^K) significantly decreased both β-arrestin-1 and -2, with the remaining five variants (A26^ECD^T, M29^ECD^T, G40^ECD^S, H88^ECD^N, and P114^ECD^A) affecting only β-arrestin-1 recruitment. Taken together, the structural analysis of variants pointed toward distinct sites within the receptor (ECD, TM1, TM2, TM3, and TM6) that can alter receptor function in a similar manner, but to a different extent, by altering one, two, or three pathways.

### Pharmacological profiling of missense variants follows real-world phenotypic traits

**Figure 6:**
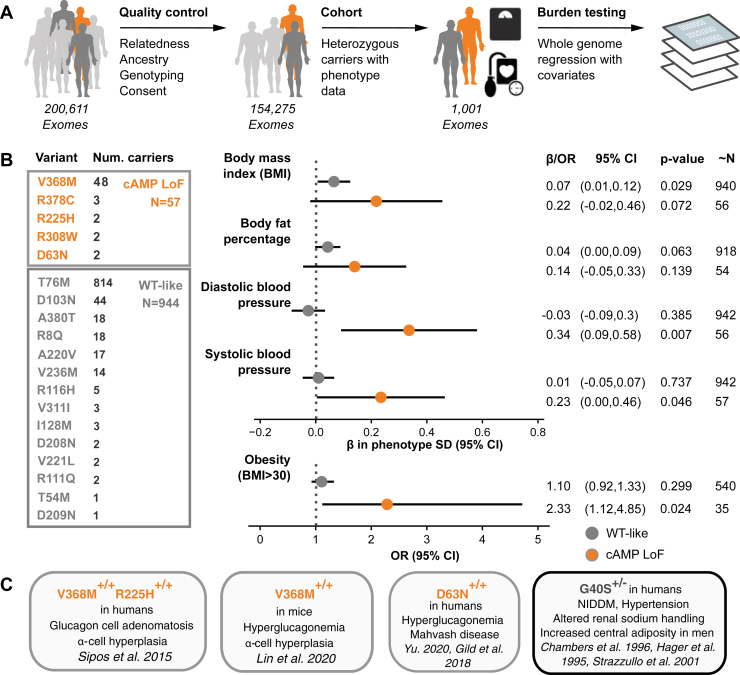
**Association of loss-of-function variants with cardiometabolic traits.***A*, from 200k Whole Exome Sequences (WES) from the UK Biobank, 154k remained after filtering out related individuals, non-Caucasian, withdrawn consent, sex chromosome aneuploidy, and outliers indicative of low genotyping quality. Of those, 1001 heterozygous carriers are used for burden testing of phenotype associations using a whole genome regression model. *B*, summary table of heterozygous carriers, and corresponding variant groups, used in burden testing. Variant groups were tested for association with quantitative and binary metabolic traits. Number of carriers included for each variant is shown. “∼N” denotes approximate number of samples for each phenotype estimated from allele frequency and total number of individuals included in each group. Note that, for quantitative traits, *x*-axis is the effect size (β) in standard deviations (SD) of original phenotype. We also tested obesity as a binary trait, for which we defined cases (N = 36,684) as BMI > 30 and controls (N = 51,315) as BMI <25. Quantitative traits were tested with linear regression and binary trait with logistic regression with Firth-correction. *C*, variants with previously linked clinical phenotypes. G40S was not included in either group given its measured impairment in β-arrestin-1 recruitment. β, Beta; NIDDM, non-insulin-dependent diabetes mellitus; OR, odds ratio (29, 31, 32, 33, 85, 86).

We assessed the prevalence and phenotypic associations of the characterized variants in Whole Exome Sequences from 200k individuals in the UK Biobank (57). In the entire cohort, we identified 1176 individuals to be carriers of pharmacologically wildtype-like variations (see Figs. 3 and 6). Based on the list of eight variants classified as significantly decreasing Gα_s_ signaling (cAMP loss of function [LoF]), we found in total 82 carriers with at least one heterozygous allele among 200,611 British samples. After quality control we retained 944 carriers of tested wildtype-like variants and 57 cAMP LoF carriers among 154,275 unrelated white British samples (Fig. 6*A*). When grouping variants by functional consequence and burden testing for association with metabolic and cardiovascular phenotypes, cAMP LoF variants suggest a near-significant trend toward higher body mass index (BMI) (effect size in SD of phenotype (β): 0.22; confidence interval [CI] = −0.02, 0.46; *p* = 0.072), body fat percentage (β: 0.14; CI = −0.05, 0.33; *p* < 0.139), diastolic blood pressure (β: 0.34; CI = 0.09, 0.58; *p* = 0.007), and systolic blood pressure (β: 0.23; CI = 0.00, 0.46; *p* = 0.046) (Fig. 6, *A* and *B*). Testing obesity as a binary trait (BMI > 30 as cases and BMI < 25 as controls) pointed toward an increased odds ratio (OR) for cAMP LoF carriers (OR: 2.33; CI = 1.12, 4.85; *p* = 0.024). Three of the five cAMP LoF variants have previously been linked to metabolic traits (Fig. 6*C*). The most frequent missense glucagon receptor variant, G40^ECD^S, has previously been linked to cardiometabolic phenotypes but is not included in the wildtype-like group, given its measured impact on β-arrestin-1 signaling.

The variants with wildtype-like signaling also showed a weak contribution to the selected binary and continuous metabolic outcomes (*e.g.*, BMI: β: 0.07; CI = 0.01012; *p* = 0.029) in the UK Biobank cohort. Overall, none of the associations reached significance (*p* < 0.005) after multiple comparison testing (Bonferroni correction) but support that *in vitro* characterization of the pharmacological parameters of the glucagon receptor are indicative for real-world clinical outcomes and that the impaired cAMP responses of the variant receptors are associated with cardiometabolic outcomes. Given the small sample size we suspect the associations may become more evident as more exome sequences are released. There are numerous glucagon receptor missense variants that were not characterized in the present study, and characterization of these may likewise uncover additional LoF variants.

## Discussion

Genetic variants can lead to disturbances throughout the life cycle of a GPCR: receptor transcription, translation, trafficking, and altered ligand interactions, which can impact signaling and interaction with associated proteins (58, 59). Several variants have been associated with receptor malfunction and have been implicated in various diseases or altered drug response. To improve our understanding of the functional and clinical impact of genetic variants, a deeper molecular and cellular understanding is required. In the present study, we identified 250 glucagon receptor missense variants in the general population (Table S1), 38 of which were selected and then investigated for altered receptor signaling properties and for glucagon binding.

As a member of class B1 GPCRs, the interaction of the glucagon receptor with glucagon follows the so-called multistep binding and activation process leading first to a preactivated ligand–receptor complex (11). For the glucagon receptor to get fully activated, it requires full coupling of a heterotrimeric G protein through an induced-fit mechanism that ultimately results in the conserved outward movement of TM6 as also seen for other class B1 receptors (16, 60). Recently, it was shown that the intracellular loops (ICLs) of the glucagon receptor are responsible for Gα_s_ protein recognition as well as specificity toward other G proteins (16). Furthermore, the extracellular parts of the TMs and ECLs are essential for interactions with the glucagon’s N-terminus and thus important for activation (60).

**Figure 7:**
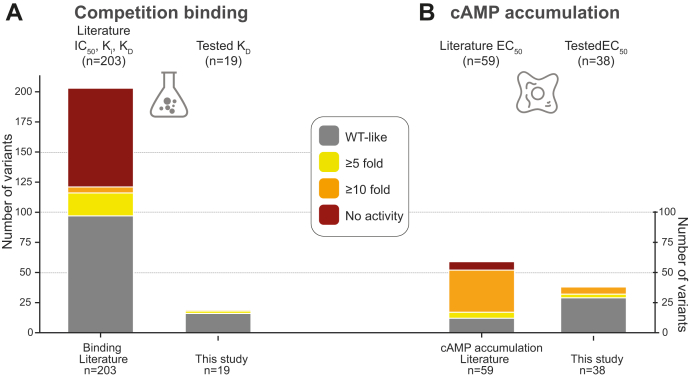
**Glucagon receptor *in vitro* mutations in the literature.***A*, *in vitro* mutations tested on binding obtained from literature annotations (n = 203, *left*) and tested in the present study (n = 19, *right*). *B*, *in vitro* mutations tested in cAMP accumulation or production experiments annotated from published studies (n = 59, *left*) and tested in the present study (n = 38, *right*). Of note, the vast majority of previously performed mutations are performed to conduct receptor structural investigations and to probe ligand interactions mostly employing Alanine or Cysteine mutations. The fraction of drastic effects is less frequently observed in human genetic variations indicating purifying selection on the most extreme phenotypes. Effects have been grouped into WT-like <5-fold difference (*gray*), ≥5-fold but <10-fold (*yellow*), ≥10-fold difference (*orange*), and no activity (*red*).

Besides Gα_s_, the glucagon receptor also interacts with Gα_q/11_ and Gα_i/o_ to initiate downstream signaling (15). In rats, for instance, Gα_q/11_-mediated calcium mobilization has been reported to be important for the inhibition of glycolysis and the stimulation of glycogenolysis (61, 62). However, in contrast to Gα_s_, Gα_q/11_ interacts weakly with the receptor (63). Thus, since Gα_s_-mediated signaling is the most established pathway for glucagon physiology (14), we focused on this in our study. Overall, we observed wildtype-like responses for 30 genetic variants. This included, for instance, D299^ECL2^N, which according to the cryo-EM structure makes a direct interaction with glucagon (16). Of the 38 mutations, eight displayed altered Gα_s_-mediated signaling (cAMP LoF), which, for the majority, involved a shift of potency, although with a much lower effect than that of other *in vitro* engineered mutations, described in the literature (Fig. 7*A*) (64). The binding affinity of glucagon to these variants, remained unaffected but showed lower B_max_, suggesting that the changes in signaling are not due to an altered glucagon binding but altered receptor activation. This information is supported by the available literature on glucagon receptor mutations (Fig. 7*B*) (64). We conclude that receptor signaling can be severely impaired by single point mutations but that negative selection prevents these from occurring as single nucleotide polymorphisms (Fig. 1).

Previous *in vivo* characterizations of missense variants in the GLP-1R and GIPR have pointed to associations between receptor impairments and clinical phenotypes. One example is the more frequently occurring variant of GIPR E354^6.53^Q (gnomAD MAF: 0.2). Homozygous carriers of this variant have lower bone mineral density, increased bone fracture risk, and slightly increased blood glucose and, at the molecular level, increased receptor internalization, leading to a long-term desensitization and impairment of the GIP system (18, 65, 66). Heterozygous carriers of the rare missense variant T149^1.44^M of the GLP-1R have a higher risk of type 2 diabetes as result of lower insulin secretion and impaired insulin sensitivity (67). In-depth molecular analysis of this variant revealed a lower binding capacity of GLP-1 as well as impaired receptor signaling capabilities (68). Several other variants (P7^ECD^L, R44^ECD^H and L260^ICL2^P) in the same study were not associated with a similar phenotype and displayed wildtype-like GLP-1 binding and receptor signaling (67). G168^1.63^S is another common variant for GLP-1R occurring in both homozygous and heterozygous carriers (gnomAD MAF: 0.23) (69, 70). This variant has no clear clinical phenotype, but treatment with liraglutide of patients with type 2 diabetes resulted in improved weight loss and lower fat mass in these individuals (69). On the other hand, obese carriers had impaired weight loss after bariatric surgery (70). As expected from these divergent observations, the molecular phenotype of this variant is wildtype-like with preserved receptor signaling and binding affinity of GLP-1 (68).

For the glucagon receptor, the metabolic phenotype of several missense variants has been investigated in cohort studies (G40^ECD^S), single-case studies (D63^ECD^N, P86^ECD^S, and the double variant R225^3.30^H-V368^6.59^M), as well as in genetically engineered animal studies (V368^6.59^M) (27, 28, 29, 30, 31, 32, 33). Table 1 provides an overview of these variants both *in vitro* and *in vivo*, with a comparison with the present study. Previously, it was highlighted that mice with deletions of the glucagon receptor and individuals with a receptor splice variant (with impaired function) have a disrupted liver-α-cell axis leading to hyperaminoacidemia, hyperglucagonemia, and α-cell hyperplasia (71, 72). Despite the impaired glucagon receptor function, and the subsequent hyperglucagonemia, blood glucose levels were normal, presumably due to a compensatory insulin secretion and normal function of the pancreatic β-cells. Another possibility could be that glucagon is unable to counterbalance insulin, but since its effect on hepatic glucose production is also impaired, blood glucose remains unchanged as observed with the glucagon receptor antagonist (73). Three of the variants in the present study (D63^ECD^N, P86^ECD^S, V368^6.59^M, and R225^3.30^H-V368^6.59^M) have been associated with hyperglucagonemia and α-cell hyperplasia in homozygous carrier(s) (Table 1) (28, 29, 30). One may also expect altered lipid metabolism in these cases, as glucagon plays a role in beta oxidation and given that glucagon receptor antagonism results in increased LDL-cholesterol and hepatic steatosis (7, 74).

**Table 1:** Summary of *in vitro* and/or *in vivo* results on published glucagon receptor missense variants and the present study

Receptor variant	Reference	Binding		Signaling		Clinical phenotype
		Experimental setup and cell type	Results	Experimental setup and cell type	Results	
G40^ECD^S	Present study	[^125^I]glucagon for 3 h at 4 °C (transient COS-7 cells)	Similar K_D_ and similar B_max_	cAMP accumulation 30-min stimulation (transient COS-7 cells)	Similar EC_50_ and E_max_	
	Siani *et al.* (27)					Central adiposity in men; 37 individuals (total 985); heterozygous
	Hager *et al.* (31)	[^125^I]glucagon for 2 h at room temperature (stable BHK cells)	3.0-fold lower K_D_, similar B_max_			Non-insulin-dependent diabetes; 13 individuals (total 1218); heterozygous
	Mukund *et al.* (82)	[^125^I]glucagon (stable HEK 293)	Similar K_D_ and B_max_	CRE-luciferase (stable HEK 293)	4.7-fold lower EC_50_	
	Hansen *et al.* (83)	[^125^I]glucagon (stable BHK cells/RIN cells)	3.5- to 6.1-fold lower K_D_, similar B_max_	cAMP accumulation (stable BHK cells/RIN cells)	2.5- to 4-fold lower pEC_50_ and lower E_max_	
	Lin *et al.* (30)	[^125^I]glucagon for 3 h at room temperature (transient CHO-K1 cells)	Similar K_D_, 1.5-fold lower B_max_	TR-FRET cAMP accumulation 40-min stimulation (transient CHO-K1 cells)	Similar EC_50_ and E_max_	
D63^ECD^N	Present study	[^125^I]glucagon for 3 h at 4 °C (transient COS-7 cells)	Increased K_D_ but very low B_max_	cAMP accumulation 30-min stimulation (transient COS-7 cells)	219.2-fold lower EC_50_ and lower E_max_	
	Gild *et al.* (33)					Hyperglucagonemia and Mahvash disease; 1 individual homozygous
P86^ECD^S	Present study	[^125^I]glucagon for 3 h at 4 °C (transient COS-7 cells)	Similar K_D_, 20-fold lower B_max_	cAMP accumulation 30-min stimulation (transient COS-7 cells)	18.6-fold lower EC_50_, similar E_max_	
	Zhou *et al.* (28)	[^125^I]glucagon for 1 h at 37 °C (transient HEK 293 cells)	29-fold lower B_max_	LANCE cAMP accumulation 60-min stimulation (transient HEK 293 cells)	9.9-fold lower EC_50_, similar E_max_	Hyperglucagonemia and ɑ-cell hyperplasia; 1 individual; homozygous
	Siu *et al.* (40)	[^125^I]glucagon for 2 h at room temperature (transient CHO-K1 cells)	No binding observed			
R225^3.30^H	Present study	[^125^I]glucagon for 3 h at 4 °C (transient COS-7 cells)	Similar K_D_, 20-fold lower B_max_	cAMP accumulation 30-min stimulation (transient COS-7 cells)	86.4-fold lower EC_50_, slightly lower E_max_	
	Lin *et al.* (30)	[^125^I]glucagon for 3 h at room temperature (transient CHO-K1 cells)	No binding observed	TR-FRET cAMP accumulation 40-min stimulation (transient CHO-K1 cells)	>10-fold lower EC_50_, similar E_max_	
R225^3.30^H-V368^6.59^M	Sipos *et al.* (29)					ɑ-Cell hyperplasia; 1 individual; homozygous
V368^6.59^M	Present study	[^125^I]glucagon for 3 h at 4 °C (transient COS-7 cells)	Similar K_D_, 3.3-fold lower B_max_	cAMP accumulation 30-min stimulation (transient COS-7 cells)	Weak activation	
	Lin *et al.* (30)	[^125^I]glucagon for 3 h at room temperature (transient CHO-K1 cells/liver cell membranes of mice)	Similar K_D_, 1.9-fold lower B_max_/similar K_D_, 4.0-fold lower B_max_	TR-FRET cAMP accumulation 40-min stimulation (transient CHO-K1 cells/liver cell membranes of mice)	2.5-fold lower EC_50_, similar E_max_/9.5-fold lower EC_50_, similar E_max_	Hyperglucagonemia and ɑ-cell hyperplasia (mice; homozygous)

Within each experiment, the receptor variant is compared with the wildtype glucagon receptor. Ligand stimulations were performed with glucagon.

Consistent with our findings, Zhou *et al.* (28) reported a 29-fold reduction in B_max_ for P86^ECD^S and an impaired potency (9.9-fold) in cAMP production. The same characteristics were found for V368^6.59^M (gnomAD MAF: 7.1E-5) by Lin *et al.* (30), who also pointed toward an impaired Gα_s_-mediated signaling for this missense variant (Table 1). Furthermore, in genetically engineered mice expressing V368^6.59^M, an improved glucose tolerance and elevated glucagon levels as well as α-cell hyperplasia and pancreas enlargement were reported, *in vivo* phenotypes comparable with that of P86^ECD^S in human (28). Hence, impaired glucagon receptor signaling for these missense variants seems to result in a disrupted liver-α-cell axis, resulting in decreased glucagon-induced hepatic amino acid catabolism and thereby leading to hyperaminoacidemia and consequently hyperglucagonemia.

For heterozygous carriers of G40^ECD^S, which is the most common missense variant in the glucagon receptor (gnomAD MAF: 0.0068), an association with non-insulin-dependent diabetes and central adiposity has been established in men *via* cohort studies in French/Sardinian patients and a large population study of men (the Olivetti Heart study) (Table 1) (27, 31). However, several groups have shown limited impact *in vitro* of G40^ECD^S, consistent with our findings where no decreased cAMP activity could be detected. Of interest, we observed a significantly decreased β-arrestin-1 signaling efficacy, which may require further investigations such as internalization studies. Moreover, no associations could be established with non-insulin-dependent diabetes among Japanese, Taiwanese, and Finnish populations (75, 76, 77). However, there could also be other mechanisms involved leading to altered receptor function, such as different ligand-receptor binding kinetics or altered signaling through pathways not yet investigated (58, 78). It is possible that G40^ECD^S might associate with adiposity, but the mutation would not be expected to be causative, given that only minor/no influence on molecular phenotype was detectable.

We also investigated the link between rare glucagon receptor missense variants and five cardiometabolic phenotypes employing 200k exome sequences available from the UK Biobank. Here, we aggregated variants by their molecular pharmacological profile rather than testing multiple individual variants for associations. This is because achieving statistical power for rare individual variants to show robust association with phenotypic traits is made difficult by the strong degree of conservation of GCGR, which results in few carriers of variants, particularly regarding highly impactful and likely deleterious variants. After pooling of individuals based on cAMP LoF variants we identified 57 carriers, which showed association to cardiometabolic trait profiles. Carriers of the wildtype-like variants are much more frequent and seem not to be associated to these traits to the same extent. This indicates that cell-based molecular phenotypes can be indicative of real-world traits. We did not observe phenotypic associations for variants impaired only in β-arrestin1/2, chiefly G40^ECD^S, which warrants further investigation of its molecular profile. Future expansions of the biobank cohort size and the molecular characterizations of additional glucagon receptor variants could become useful to increase power and to expand the investigation toward additional less frequent phenotypes.

In conclusion, we present multiple missense variants in the human glucagon receptor gene and their consequences for molecular pharmacological parameters, thereby providing a link between molecular understanding and clinical phenotypes. We highlight that the glucagon receptor has fewer missense variants, lower allele diversity, and fewer variants with trait association than other class B1 receptors, and we suggest that rare variants are associated with cardiometabolic outcomes. Future studies on the physiological and clinical phenotypes of variants with impaired activation and/or altered binding capacities are warranted. Such studies can take advantage of more specific cohorts with focus on endocrinology and metabolism in conjunction with *in vivo* pharmacological characterizations of a broader set of genetic variations. We hope that the findings presented here will fuel further research in the targeted data integration spanning genetics, evolutionary biology, structural studies, pharmacology, and clinical epidemiology both for the glucagon receptor and other GPCRs.

## Experimental procedures

### Materials

The human glucagon receptor was inserted into the pcDNA3.1 plasmid (GenBank accession number: NM_000160 and NP_000151). Human glucagon(1–29) was purchased from Caslo ApS (Lyngby). COS-7 and HEK 293 cells were purchased from ATTC. COS-7 cell medium was prepared in-house, whereas HEK 293 cell medium was bought from Thermo Scientific. [^125^I]glucagon was received from Novo Nordisk A/S (Bagsværd). Polyethylenimine (PEI) was purchased from Polysciences Inc. Coelenterazine h was bought from Nanolight Technologies. Other chemicals were purchased from standard commercial sources.

### Site-directed mutagenesis

Point mutations were introduced in the receptor according to a quick-change protocol, using predesigned primers (Table S4). These primers were created based on the following criteria: 25 to 30 nucleotides long, G and C base pairs at the 3′ and 5′ ends, predicted melting temperature between 75 and 90 °C and a GC content between 50% and 80%. Next, a PCR master mix was prepared, consisting of 0.2 μM dNTPs (Invitrogen), 0.06 U/ml Pfu polymerase (Promega), 1× Pfu buffer (Promega), 0.4 μM of both forward and reverse primer (Tag Copenhagen A/S), and 0.05 μg glucagon receptor wildtype in pcDNA3.1. The PCR reaction was executed according to a two-phase protocol on a Master Cycle PCR (Eppendorf): the first phase consisted of 30 cycles at 60 °C for 30 s, 55 °C for 15 s, 72 °C for 30 s, and 95 °C for 5 min. The second phase involved 18 cycles at 63 °C and at 68 °C for 1 s, after which the reaction was terminated and held at 4 °C. Digestion of methylated DNA was performed with 20 U/μl Dpn1 (New England Biolabs Inc). After transformation and DNA purification, the mutant receptors were validated using Sanger sequencing (Eurofins Scientific).

### Cell culturing and transfection

COS-7 cells were cultured in DMEM (Dulbecco’s modified Eagle’s medium) 1885 medium, containing 3.9 g/l NaHCO_3_, and supplemented with 10% fetal bovine serum, 1% l-glutamine, and 1% penicillin (180 U/ml)/streptomycin (45 μg/ml). HEK 293 cells were cultured in DMEM supplemented with 10% fetal bovine serum, 1% penicillin (180 units/ml)/streptomycin (45 μg/ml), and incubated at 10% CO_2_, 95% air humidity, and 37 °C. All cells were subcultured twice a week, after the release with 1% Trypsin, and incubated at 37 °C, 10% CO_2_, and 95% air humidity. The cell viability was checked microscopically, and their density was measured using the NucleoCounter SCC-100 (Chemometec). All cells were tested and were free from mycoplasma contamination.

COS-7 cells (1.2–1.5 × 10^6^ cells/flask) were seeded in 25-cm^2^ flasks before a calcium phosphate transfection was performed. Ten micrograms of human wildtype or mutant glucagon receptor (or pcDNA3.1) in 120 μl of Tris-EDTA (TE) buffer (10 mM Tris-HCl, 2 mM EDTA-Na_2_, pH 7.5) was mixed with 15 μl of CaCl_2_ and then titrated into 120 μl 2× Hepes-buffered saline (HBS) buffer (280 mM NaCl, 50 mM Hepes, 1.5 mM Na_2_HPO_4_, pH 7.2) to perform a transient transfection of COS-7 cells. The mixture was incubated for 45 min at room temperature, before it was added dropwise to the cells together with 2 mg/ml chloroquine. After 5 h, the transfection was terminated by replacing the medium with fresh supplemented DMEM 1885 NaHCO_3_ medium.

HEK 293 cells were seeded in Costar tissue culture treated 6-well plates (500,000 cells/well, Corning Inc) 1 day prior to a PEI transfection. Cell transfection was performed by mixing 2.34 μg of PEI (to obtain DNA/PEI ratio 1:2) with 182.2 μl nonsupplemented DMEM, followed by a 5-min incubation at RT. Afterward, 0.33 μg wildtype glucagon receptor or mutant receptor, 0.042 μg Rluc8-Arr2-Sp1 or Rluc8-Arr3-Sp2, and 0.8 μg mem-citrine-SH3 were mixed with PEI/DMEM and incubated for 15 min at RT before the mixture was added dropwise to the cells. The transfection was stopped after 24 h by replacing the transfection medium with 3 ml of fresh supplemented DMEM, followed by an incubation for 1 day at 10% CO_2_, 95% air humidity, and 37 °C.

### cAMP assay

The DiscoverX HitHunter cAMP assay was performed according to the manufacturer’s protocol (DiscoverX). One day before the assay, the transiently transfected COS-7 cells were seeded into a 96 white CulturPlate (35,000 cells/well, PerkinElmer). On the assay day, the cells were washed with 1× HBS and subsequently incubated in 100 μl of 1 mM 3-isobutyl-1-methylxanthine diluted in 2× HBS for 30 min at 37 °C. Five microliters of glucagon(1–29) (concentrations ranged from 1 pM to 10 nM) was then added, and the plate was incubated for 30 min at 37 °C. After the incubation, the assay medium was removed from the plate; the cells were washed with 30 μl PBS and afterward treated with 40 μl ED/Lysis/CL and 10 μl cAMP antibodies for 60 min, before 40 μl enzyme acceptor solution was added. After a 3-h incubation in the dark, the accumulation of cAMP was measured as luminescence using the PerkinElmer EnVision 2104 Multilabel Microplate Reader.

### Bioluminescence resonance energy transfer β-arrestin 1/2 recruitment assay

The β-arrestin 1/2 recruitment assay was performed as described (17). Briefly, the bioluminescence resonance energy transfer assay was conducted by washing the cells with 2 ml PBS followed by resuspension in 2 ml PBS + 1% glucose (0.5 M). Next, the cells were aliquoted (85 μl/well) into white PerkinElmer 96-well plates. Coelenterazine h, 5 mM, was diluted with PBS to a concentration of 50 μM and kept in the dark. A volume of 10 μl/well of coelenterazine h (final concentration 5 μM) solution was added, and the plate was incubated for 10 min. The reaction was initiated by adding glucagon(1–29) in concentrations ranging from 0.1 nM to 10 μM. After a 30-min incubation at RT the luminescence (ratio of 535 nm over 480 nm emission) was measured by a PerkinElmer EnVision 2104 Multilabel Microplate Reader.

### Homologous competition binding assay

One day prior to the assay, the transfected COS-7 cells were seeded in a 96 white CulturPlate (5000–7500 cells/well for the wildtype glucagon receptor and 5000–45,000 cells/well for missense variants). On the assay day, the cells were washed twice with binding buffer (50 mM Hepes buffer (pH 7.2), 1 mM CaCl_2_, 5 mM MgCl_2_, 0.5% (w/v) BSA) and incubated for 15 min at 5 °C. After the addition of cold glucagon (concentration ranging from 0.1 nM to 1 μM), 22.99 ± 2.50 pM [^125^I]glucagon was added, and the plate was incubated for 3 h at 4 °C. The reaction was terminated by washing two times with ice-cold binding buffer, and the cells were subsequently lysed with 200 mM NaOH containing 1% SDS. The gamma radiation intensity was measured with a PerkinElmer 2470 Wizard2 Automatic Gamma Counter.

### Data analysis

The nonlinear regression curve fitting program GraphPad 9 was used to analyze the data and to obtain EC_50_, E_max_, AUC, IC_50_, K_D_, and B_max_ values (GraphPad software). All sigmoidal curves were fitted with a Hill slope of 1 (cAMP accumulation) or −1 (homologous competition binding).

The B_max_ (the total density of receptors in the sample) of the wildtype and mutations were calculated from competitive binding curves according to the following equation (79):

$${\text{B}}_{\text{max}}=\frac{{\text{B}}_{0}*{\text{IC}}_{50}}{\left[\text{L}\right]}$$

wherein B_0_ is the total specific binding and [L] is the ligand concentration.

The equilibrium dissociation constant (K_D_) was obtained by using the following equation (79):

$${\text{K}}_{\text{D}}={\text{IC}}_{50}-\left[\text{L}\right]$$

Statistical significance was addressed by an ordinary one-way ANOVA for E_max_, EC_50_, and AUC in cAMP accumulation and β-arrestin 1/2 recruitment. For binding experiments, B_max_ and K_D_ were also assessed by an ordinary one-way ANOVA. Two-way ANOVA was used for dose-dependent comparison in cAMP accumulation with V368^6.59^M. The definition of statistical significance was *p* < 0.05.

### Genetic variation data, disease mutations, summary genetic associations, literature annotation data, and phenotype associations

We considered the location of GCGR to be chr17: 81,804,150 to 81,814,008 forward strand, and all variant alleles are in reference to the Ensembl canonical transcript, ENST00000400723.8. We obtained natural genetic variation data for all class B1 GPCRs from the gnomAD, which is compiled data on 125,748 exome- and 15,708 whole-genome sequences from human sequencing studies from seven distinct human populations (accessed July 16, 2020) (34). In addition, we retrieved data on the expected number of missense variants, *i.e.*, in the absence of selection, and the actual observed numbers of missense variations (34). Briefly, the prediction of the level of expected variation under neutrality is based on a mutational model incorporating methylation and base-level coverage correction and uses the loss-of-function transcript effect estimator (LOFTEE). We calculated an allele diversity metric per missense variant position normalized by the receptor coding length to estimate and compare allele diversity not just by the number of variant positions but also the allele frequency of a given variation.

Disease mutations were retrieved from the DisGeNET knowledge platform for disease genomics (36), aggregating data from various platforms, covering more than 117,000 disease-associated genomic variants for nearly all genes. The variants were mapped to genes using DisGeNET’s “variant_to_gene_mappings.tsv.” We included all unique variants mapped to a gene including all variant types and irrespective of the number of disease associations a variant can manifest.

We analyzed data on genetic associations for 778 traits of 452,264 UK Biobank participants of European ancestry as described and collected on GeneATLAS (39). We extracted gene location–specific trait associations (*p* ≤ 0.001) for all class B1 genes and aggregated the number of unique variants with trait associations per gene.

We collected data on previously engineered glucagon receptor *in vitro* mutations from the GPCRdb (80) with reported fold-change effect values on ligand affinity and/or potency. We restricted binding and cAMP accumulation values to studies using glucagon as a ligand but allowed investigations on orthologous receptors from related species such as mouse, rat, hamster, dog, pig, and rhesus macaque if the amino acid was identical to the corresponding generic residue position in the human receptor.

To perform phenotype associations of GCGR LoF missense variants we obtained 200k exome sequences pVCF files from the UK Biobank. We restricted our analysis to Caucasian samples (field #22006), filtered outliers for heterozygosity or missing rate (#22027) as well as sex chromosome aneuploidy (#22019). We furthermore filtered samples with a KING kinship coefficient (UKB provided) above 0.088. We quality controlled samples with mean call rate <0.97, mean read depth (DP) < 15, and mean genotype quality <50 and filtered out variants with HWE *p* < 10e-15 and mean GQ < 20. Calls were filtered for GQ < 20, DP < 10, and allele balance (AB, for each allele) < 0.25 (https://hail.is). Burden testing was performed with Regenie v2.2.4 (81) with cAMP LOF and WT-like variants as masks. Step 1 was run as per the recommendation for UKB data with covariates sex, age, age2, age∗sex, age2∗sex, and the first 20 PCs (#22009). Association testing was done using linear regression for rank-based inverse normal transformed quantitative traits, namely, BMI (#21001), Body fat percentage (#23099), Diastolic blood pressure (either #94 or #4079), and Systolic blood pressure (##93 or #4080). Logistic regression with Firth correction was used to test binary trait: obesity (BMI > 30), controls (BMI < 25).

## Data availability

Data are available upon request by contacting the corresponding authors.

## Supporting information

This article contains supporting information.

## Conflict of interest

The authors declare that they have no conflicts of interest with the contents of this article.
