# Supplementary material for: Molecular and in vivo phenotyping of missense variants of the human glucagon receptor
Source: J Biol Chem. 2021 Nov 19;298(2):101413. doi: 10.1016/j.jbc.2021.101413 (PMC8829087; doi:10.1016/j.jbc.2021.101413)
Supplement: Supplemental Figure S1 and Tables S1–S4 [file mmc1.pdf]

Supplementary Table 1 | gnomAD and literature found human missense variants in the glucagon receptor

Missense variant	Location	GnomAD MAF	Reference id	GnomAD Allele count	Number of homozygotes	Transcript Consequence
P2S	ECD	7.2E-06	rs1374465504	1	0	c.4C>T
**P3S**	ECD	7.2E-06	rs746335439	1	0	c.7C>T
P3T	ECD	7.2E-06	rs746335439	1	0	c.7C>A
P3L	ECD	7.2E-06	rs1050897506	1	0	c.8C>T
Q5P	ECD	2.9E-05	rs1271734930	4	0	c.14A>C
**R8Q**	ECD	2.9E-05	rs773994205	4	0	c.23G>A
P9L	ECD	3.2E-05	rs1233390237	1	0	c.26C>T
L13V	ECD	7.4E-06	rs924505906	1	0	c.37T>G
L17P	ECD	2.9E-05	rs953530197	5	0	c.50T>C
A18D	ECD	1.2E-05	rs1335228562	2	0	c.53C>A
C19W	ECD	1.5E-05	rs1054447666	2	0	c.57C>G
P24H	ECD	7.1E-06	rs1323911527	1	0	c.71C>A
**A26T**	ECD	6.4E-05	rs770131289	11	0	c.76G>A
**M29T**	ECD	1.7E-05	rs747611271	3	0	c.86T>C
D30H	ECD	7.1E-06	rs1263930997	1	0	c.88G>C
D30G	ECD	7.1E-06	rs1480166504	1	0	c.89A>G
**G40S**	ECD	6.8E-03	rs1801483	1170	5	c.118G>A
G40A	ECD	7.1E-06	rs1158418305	1	0	c.119G>C
**D41E**	ECD	1.4E-05	rs760232106	2	0	c.123C>A
H45Y	ECD	1.4E-05	rs984272223	2	0	c.133C>T
S48N	ECD	7.1E-06	rs1364365831	1	0	c.143G>A
P52T	ECD	7.1E-06	rs1331669976	1	0	c.154C>A
P52S	ECD	7.1E-06	rs1331669976	1	0	c.154C>T
**T54M**	ECD	1.5E-04	rs373371585	21	0	c.161C>T
**R60T**	ECD	7.0E-06	rs561472081	1	0	c.179G>C
F62L	ECD	7.0E-06	rs1381720436	1	0	c.184T>C
**D63N**	ECD	3.5E-05	rs1219737977	5	0	c.187G>A

D63A	ECD	7.0E-06	rs1283204989	1	0	c.188A>C
P69L	ECD	1.4E-05	rs576194179	2	0	c.206C>T
A73T	ECD	4.6E-05	rs868431560	8	0	c.217G>A
**T76M**	ECD	1.3E-03	rs189736697	232	1	c.227C>T
I79V	ECD	2.3E-05	rs1038824634	4	0	c.235A>G
P82S	ECD	7.0E-06	rs1378394827	1	0	c.244C>T
**P86S**[a]	ECD				1	c.256C>T
**H88N**	ECD	1.7E-05	rs755602452	3	0	c.262C>A
H93Q	ECD	7.1E-06	rs1222169977	1	0	c.279C>A
R94C	ECD	3.5E-05	rs552010052	6	0	c.280C>T
R94H	ECD	1.4E-05	rs1022912945	2	0	c.281G>A
**V96E**	ECD	7.1E-06	rs756454904	1	0	c.287T>A
V96M	ECD	2.1E-05	rs1240225006	3	0	c.286G>A
V96A	ECD	7.1E-06	rs756454904	1	0	c.287T>C
K98R	ECD	3.2E-05	rs1253073377	1	0	c.293A>G
R99S	ECD	3.2E-05	rs1193513499	1	0	c.297A>T
G101R	ECD	2.1E-05	rs373865736	3	0	c.301G>A
**D103N**	ECD	1.2E-04	rs779580966	20	0	c.307G>A
D103H	ECD	3.2E-05	rs779580966	1	0	c.307G>C
G104S	ECD	7.1E-06	rs1377157654	1	0	c.310G>A
R108H	ECD	2.8E-05	rs1312508282	4	0	c.323G>A
P110S	ECD	1.7E-05	rs1043477675	3	0	c.328C>T
P110H	ECD	1.4E-05	rs1228449257	2	0	c.329C>A
**R111Q**	ECD	2.3E-05	rs28522411	4	0	c.332G>A
R111W	ECD	1.7E-05	rs1272192802	3	0	c.331C>T
G112E	ECD	2.9E-05	rs1258411324	5	0	c.335G>A
Q113R	ECD	3.2E-05	rs1372556587	1	0	c.338A>G
**P114A**	ECD	1.2E-03	rs5385	206	0	c.340C>G
P114T	ECD	7.1E-06	rs5385	1	0	c.340C>A
W115S	ECD	7.1E-06	rs1235960477	1	0	c.344G>C
**R116H**	ECD	1.4E-05	rs567794794	2	0	c.347G>A
S119T	ECD	6.4E-05	rs1271662476	2	0	c.355T>A
Q120R	ECD	3.2E-05	rs1305456353	1	0	c.359A>G

**M123I**	ECD	2.8E-05	rs780610679	4	0	c.369G>A
D124E	ECD	2.9E-05	rs745508664	5	0	c.372T>G
**G125C**	ECD	9.2E-05	rs529078630	13	0	c.373G>T
E126K	ECD	2.8E-05	rs889041091	4	0	c.376G>A
**I128M**	ECD	9.8E-05	rs181081681	17	0	c.384T>G
V130F	ECD	3.2E-05	rs1008826316	1	0	c.388G>T
**A135T**	TM1	1.7E-05	rs573421080	3	0	c.403G>A
M137I	TM1	1.4E-05	rs947568418	2	0	c.411G>T
S140N	TM1	7.1E-06	rs754197868	1	0	c.419G>A
F141L	TM1	7.1E-06	rs1478580187	1	0	c.423C>A
V143A	TM1	7.1E-06	rs1157583633	1	0	c.428T>C
Y145N	TM1	3.2E-05	rs1269119839	1	0	c.433T>A
T146R	TM1	7.1E-06	rs1420954588	1	0	c.437C>G
A155T	TM1	7.1E-06	rs755419233	1	0	c.463G>A
**A159T**	TM1	2.9E-05	rs758874938	5	0	c.475G>A
G165S	TM1	3.2E-05	rs1222914520	1	0	c.493G>A
L166R	TM1	3.2E-05	rs1241628118	1	0	c.497T>G
S167R	ICL1	2.8E-05	rs969354907	4	0	c.501C>G
T172I	TM2	6.3E-05	rs1276893614	11	0	c.515C>T
R173C	TM2	3.5E-05	rs867133127	6	0	c.517C>T
R173L	TM2	1.4E-05	rs867051527	2	0	c.518G>T
R173H	TM2	7.1E-06	rs867051527	1	0	c.518G>A
A175T	TM2	7.1E-06	rs1482107974	1	0	c.523G>A
A178V	TM2	5.8E-05	rs571642081	10	0	c.533C>T
A178T	TM2	6.4E-05	rs1487695265	2	0	c.532G>A
N179T	TM2	1.7E-05	rs1027790160	3	0	c.536A>C
N179D	TM2	7.0E-06	rs756243536	1	0	c.535A>G
A182V	TM2	1.4E-05	rs550681728	2	0	c.545C>T
K187E	TM2	7.0E-06	rs1399499869	1	0	c.559A>G
S189I	TM2	7.0E-06	rs1296860048	1	0	c.566G>T
V191M	TM2	7.0E-06	rs1044985218	1	0	c.571G>A
T200I	TM2	1.4E-05	rs1234915081	2	0	c.599C>T
T200A	TM2	3.2E-05	rs1310818080	1	0	c.598A>G

T200S	TM2	7.0E-06	rs1310818080	1	0	c.598A>T
R201H	TM2	7.0E-06	rs1213878850	1	0	c.602G>A
Q204R	ECL1	7.0E-06	rs1488610176	1	0	c.611A>G
Q204H	ECL1	7.1E-06	rs1190563230	1	0	c.612G>T
Q204H	ECL1	7.1E-06	rs1190563230	1	0	c.612G>C
K205N	ECL1	7.1E-06	rs1264921069	1	0	c.615A>T
**I206T**	ECL1	2.1E-05	rs1434718027	3	0	c.617T>C
**D208N**	ECL1	1.4E-05	rs1378474345	2	0	c.622G>A
D208G	ECL1	7.1E-06	rs1419135924	1	0	c.623A>G
**D209N**	ECL1	7.1E-06	rs754634656	1	0	c.625G>A
D209E	ECL1	1.4E-05	rs1401075459	2	0	c.627C>A
L210F	ECL1	7.1E-06	rs1293495447	1	0	c.628C>T
W215R	ECL1	7.1E-06	rs1301503565	1	0	c.643T>C
L216F	ECL1	7.1E-06	rs1375779374	1	0	c.646C>T
**A220V**	TM3	1.4E-05	rs762346408	2	0	c.659C>T
**V221L**	TM3	9.9E-05	rs753414714	14	0	c.661G>C
V221A	TM3	7.1E-06	rs1380723289	1	0	c.662T>C
**R225H**	TM3	2.9E-04	rs371217388	41	2	c.674G>A
A228V	TM3	2.9E-05	rs758161776	5	0	c.683C>T
A228T	TM3	1.7E-05	rs373598736	3	0	c.682G>A
V229L	TM3	6.9E-06	rs561479377	1	0	c.685G>C
V229G	TM3	6.9E-06	rs1423606565	1	0	c.686T>G
M231V	TM3	6.8E-06	rs528738808	1	0	c.691A>G
**V236M**	TM3	1.7E-04	rs781413720	31	0	c.706G>A
**Y239C**	TM3	2.0E-05	rs1297475505	3	0	c.716A>G
C240F	TM3	2.0E-05	rs975953807	3	0	c.719G>T
V244A	TM3	6.5E-06	rs1276798780	1	0	c.731T>C
L249V	TM3	6.6E-06	rs775595105	1	0	c.745C>G
N251K	TM3	3.9E-05	rs749623311	7	0	c.753C>A
N251I	TM3	1.1E-05	rs1242988043	2	0	c.752A>T
N251S	TM3	3.2E-05	rs1242988043	1	0	c.752A>G
G254R	TM3	1.7E-05	rs1358089389	3	0	c.760G>C
L258P	ICL2	7.0E-06	rs1235094308	1	0	c.773T>C

E260Q	ICL2	7.0E-06	rs999148337	1	0	c.778G>C
E260K	ICL2	7.0E-06	rs999148337	1	0	c.778G>A
S262R	TM4	2.1E-05	rs776062273	3	0	c.786C>G
S262R	TM4	7.0E-06		1	0	c.784A>C
L266I	TM4	7.0E-06	rs1454402740	1	0	c.796C>A
Y267C	TM4	2.1E-05	rs758992810	3	0	c.800A>G
I270V	TM4	2.9E-05	rs1037221263	5	0	c.808A>G
G271S	TM4	1.4E-05	rs995806123	2	0	c.811G>A
G271A	TM4	1.4E-05	rs550972775	2	0	c.812G>C
V279I	TM4	3.2E-05	rs1439311133	1	0	c.835G>A
V280I	TM4	1.0E-04	rs548905669	18	0	c.838G>A
V285I	TM4	7.0E-06	rs1218451512	1	0	c.853G>A
C287Y	TM4	4.6E-05	rs781285550	8	0	c.860G>A
E290K	TM4	2.8E-04	rs200674527	49	0	c.868G>A
E290A	TM4	1.2E-05	rs1440223162	2	0	c.869A>C
V292I	ECL2	7.0E-06	rs908475371	1	0	c.874G>A
V292F	ECL2	3.2E-05	rs908475371	1	0	c.874G>T
V292G	ECL2	7.0E-06	rs1168349939	1	0	c.875T>G
S297N	ECL2	7.1E-06	rs575272671	1	0	c.890G>A
**D299N**	ECL2	2.1E-05	rs761720188	3	0	c.895G>A
M301T	ECL2	7.1E-06	rs1214298781	1	0	c.902T>C
**L307P**	TM5	4.6E-05	rs373391581	8	0	c.920T>C
**R308W**	TM5	8.7E-05	rs1209087661	15	0	c.922C>T
R308L	TM5	7.1E-06	rs1266924744	1	0	c.923G>T
P310L	TM5	7.1E-06	rs1195738034	1	0	c.929C>T
**V311I**	TM5	3.5E-05	rs377519283	6	0	c.931G>A
I315M	TM5	1.4E-05	rs1454621277	2	0	c.945C>G
F319L	TM5	2.8E-05	rs774799702	4	0	c.957C>A
F319Y	TM5	3.2E-05	rs1223567514	1	0	c.956T>A
F320S	TM5	1.7E-05	rs1049700509	3	0	c.959T>C
F322L	TM5	2.5E-04	rs762562348	44	0	c.966C>G
F322L	TM5	1.4E-05	rs1256072758	2	0	c.964T>C
V323I	TM5	6.4E-05	rs151274323	11	0	c.967G>A

R324C	TM5	1.7E-05	rs371422062	3	0	c.970C>T
R324H	TM5	1.7E-05	rs1172127702	3	0	c.971G>A
I325V	TM5	3.2E-05	rs1453657226	1	0	c.973A>G
V326I	TM5	2.9E-05	rs538739071	5	0	c.976G>A
L328Q	TM5	7.1E-06	rs1310958309	1	0	c.983T>A
L328P	TM5	7.1E-06	rs1310958309	1	0	c.983T>C
V330M	TM5	3.2E-05	rs371777245	1	0	c.988G>A
L333R	TM5	7.1E-06	rs1338189422	1	0	c.998T>G
R334Q	TM5	3.5E-05	rs922855912	6	0	c.1001G>A
R334W	TM5	1.7E-05	rs976902380	3	0	c.1000C>T
A335T	TM5	3.2E-05	rs954105221	1	0	c.1003G>A
R336Q	ICL3	4.7E-04	rs531523452	66	2	c.1007G>A
R336W	ICL3	2.1E-05	rs568466743	3	0	c.1006C>T
H339R	ICL3	7.1E-06	rs1486905557	1	0	c.1016A>G
T341A	ICL3	7.1E-06	rs936322272	1	0	c.1021A>G
R346W	TM6	9.2E-05	rs766354711	13	0	c.1036C>T
R346Q	TM6	1.4E-05	rs1419839154	2	0	c.1037G>A
A348G	TM6	7.1E-06	rs1211413966	1	0	c.1043C>G
T353A	TM6	2.1E-05	rs1361576793	3	0	c.1057A>G
L358V	TM6	5.8E-05	rs748764343	10	0	c.1072C>G
V360I	TM6	7.1E-06	rs978118361	1	0	c.1078G>A
**E362K**	TM6	1.4E-05	rs1441715310	2	0	c.1084G>A
E362Q	TM6	7.1E-06	rs1441715310	1	0	c.1084G>C
A366T	TM6	1.2E-04	rs370541643	17	0	c.1096G>A
**V368M**	ECL3	7.1E-05	rs771824180	10	1	c.1102G>A
T369M	ECL3	1.4E-05	rs947813160	2	0	c.1106C>T
E371K	ECL3	7.1E-06	rs1335016020	1	0	c.1111G>A
A373T	ECL3	1.7E-05	rs1439063828	3	0	c.1117G>A
A373S	ECL3	7.1E-06	rs1439063828	1	0	c.1117G>T
A373V	ECL3	7.1E-06	rs1182403822	1	0	c.1118C>T
G375S	TM7	3.2E-05	rs1313639238	1	0	c.1123G>A
T376N	TM7	2.3E-05	rs372612289	4	0	c.1127C>A
T376S	TM7	7.1E-06	rs372612289	1	0	c.1127C>G

**R378C**	TM7	1.7E-05	rs1474100584	3	0	c.1132C>T
R378H	TM7	7.1E-06	rs1166903170	1	0	c.1133G>A
**A380T**	TM7	4.1E-05	rs377211796	7	0	c.1138G>A
D385N	TM7	7.1E-06	rs1355452290	1	0	c.1153G>A
F391L	TM7	5.7E-05	rs752844034	8	0	c.1173C>A
L395P	TM7	4.9E-05	rs1396277104	7	0	c.1184T>C
V396E	TM7	7.0E-06	rs1287425806	1	0	c.1187T>A
V398I	TM7	2.1E-05	rs747664695	3	0	c.1192G>A
L399P	TM7	3.2E-05	rs1458636561	1	0	c.1196T>C
V407M	H8	7.3E-06	rs1253368710	1	0	c.1219G>A
S409W	H8	4.7E-05	rs527317042	8	0	c.1226C>G
S409L	H8	1.2E-05	rs527317042	2	0	c.1226C>T
S409P	H8	7.2E-06	rs1187609450	1	0	c.1225T>C
R412L	H8	1.2E-04	rs775647518	20	0	c.1235G>T
R412W	H8	5.9E-05	rs552133484	10	0	c.1234C>T
R412Q	H8	7.2E-06	rs775647518	1	0	c.1235G>A
R413W	H8	5.0E-05	rs757894502	7	0	c.1237C>T
R413Q	H8	7.2E-06	rs887645895	1	0	c.1238G>A
R414H	H8	7.0E-04	rs538504452	119	1	c.1241G>A
H416R	H8	4.3E-04	rs137897678	74	1	c.1247A>G
H416L	H8	3.4E-04	rs137897678	59	0	c.1247A>T
H416Y	H8	7.2E-06	rs1357168725	1	0	c.1246C>T
R417H	H8	8.6E-05	rs893269823	12	0	c.1250G>A
R417C	H8	1.8E-05	rs1306988530	3	0	c.1249C>T
R419S	H8	9.3E-05	rs558491682	13	0	c.1255C>A
R419L	H8	2.9E-05	rs769673542	5	0	c.1256G>T
R419H	H8	2.3E-05	rs769673542	4	0	c.1256G>A
R419C	H8	7.1E-06	rs558491682	1	0	c.1255C>T
V423L	H8	3.2E-05	rs1222479831	1	0	c.1267G>C
L424P	H8	3.2E-05	rs1487455874	1	0	c.1271T>C
R428Q	H8	4.7E-04	rs61734385	81	1	c.1283G>A
R428W	H8	1.2E-05	rs1171829126	2	0	c.1282C>T
T430I	C-term	7.1E-06	rs1311017995	1	0	c.1289C>T

H433P	C-term	7.1E-06	rs1377024369	1	0	c.1298A>C
H433R	C-term	7.1E-06	rs1377024369	1	0	c.1298A>G
S438L	C-term	5.0E-04	rs545914836	87	1	c.1313C>T
G440S	C-term	8.7E-05	rs183571856	15	0	c.1318G>A
G440R	C-term	7.1E-06	rs183571856	1	0	c.1318G>C
G442S	C-term	2.1E-05	rs1021042497	3	0	c.1324G>A
G442V	C-term	7.1E-06	rs188519815	1	0	c.1325G>T
P443S	C-term	1.4E-05	rs1211330325	2	0	c.1327C>T
E447K	C-term	1.4E-05	rs981152634	2	0	c.1339G>A
E447D	C-term	7.1E-06	rs1187930393	1	0	c.1341G>C
G451R	C-term	7.1E-06	rs1453320474	1	0	c.1351G>A
R452K	C-term	7.1E-06	rs761153740	1	0	c.1355G>A
G455V	C-term	1.4E-05	rs1414985683	2	0	c.1364G>T
G455R	C-term	7.1E-06	rs1396876164	1	0	c.1363G>C
S456T	C-term	5.8E-05	rs766891858	10	0	c.1367G>C
Q457R	C-term	1.7E-05	rs754349828	3	0	c.1370A>G
D458H	C-term	2.3E-03	rs138492107	405	1	c.1372G>C
D458E	C-term	7.1E-06	rs1365987180	1	0	c.1374T>G
A461V	C-term	5.4E-04	rs13306383	93	3	c.1382C>T
T463I	C-term	7.1E-06	rs1220319020	1	0	c.1388C>T
L469F	C-term	2.8E-05	rs780849172	4	0	c.1405C>T
A473V	C-term	2.3E-05	rs975232367	4	0	c.1418C>T
S475R	C-term	3.2E-05	rs1283696557	1	0	c.1425C>A
P476L	C-term	4.2E-05	rs745618150	6	1	c.1427C>T

MAF is the minor allele frequency found in the gnomAD. a Literature (28). Selected residues for functional characterization are highlighted in bold.

Supplementary Table 2 | Functional characterization of selected missense variants in the glucagon receptor

Variant	cAMP accumulation											B-arrestin 1 recruitment											B-arrestin 2 recruitment										
	Log EC50	SEM	p-value	Emax % WT	SEM	p-value	AUC % WT	SEM	p-value	Fmut	n	Log EC50	SEM	p-value	Emax % WT	SEM	p-value	AUC % WT	SEM	p-value	Fmut	n	Log EC50	SEM	p-value	Emax % WT	SEM	p-value	AUC % WT	SEM	p-value	Fmut	n
**WT**	-10,18	0,09		100,0	0,00		100,0	11		1	33	-6,95	0,07		100,0			100,0	12,60		1	31	-7,02	0,08		100,0	0,00		100,0	15,49		1,0	31
**P3S**	-9,93	0,26	0,999	128,0	10,31	0,489	114,4	23	0,999	1,64	4	-7,03	0,09	>0,9999	103,5	21,80	1,000	109,1	22,30	1,000	0,98	5	-7,02	0,14	>0,9999	139,9	30,16	0,340	144,1	18,90	0,863	0,9	5
**R8Q**	-10,15	0,14	>0,9999	104,3	13,41	1,000	103,8	9	1,000	0,59	3	-6,89	0,11	>0,9999	72,9	12,73	0,650	80,0	15,58	0,999	1,4	5	-6,97	0,05	>0,9999	124,4	22,53	0,984	124,1	17,87	0,999	0,9	5
**A26T**	-9,89	0,20	0,999	110,6	8,34	0,999	103,7	9	1,000	1,13	3	-7,06	0,16	1,000	56,7	5,21	0,024	67,8	15,33	0,981	1,04	5	-7,04	0,10	>0,9999	105,3	31,90	1,000	106,6	23,78	1,000	0,8	5
**M29T**	-9,33	0,41	0,599	102,0	17,88	1,000	65,2	13	0,992	5,67	3	-6,37	0,15	0,999	52,1	9,82	0,007	49,2	11,94	0,363	5,41	5	-6,50	0,19	0,999	81,1	28,38	0,999	61,1	16,85	0,961	3,2	5
**G40S**	-10,06	0,33	1,000	120,6	22,45	0,985	113,2	16	1,000	0,92	3	ND	ND	ND	41,2	15,84	0,000	44,1	16,13	0,203	ND	5	-6,86	0,11	1,000	84,8	19,51	0,999	75,2	15,63	0,999	1,2	5
**D41E**	-10,22	0,11	>0,9999	100,1	7,60	>0,9999	104,7	9	1,000	0,54	3	-7,03	0,07	>0,9999	111,7	25,09	0,999	122,5	25,10	0,999	1,06	5	-7,29	0,10	1,000	90,2	13,57	0,999	112,4	16,51	1,000	0,3	5
**T54M**	-10,02	0,26	1,000	103,0	9,74	1,000	97,7	11	>0,999	1,15	3	-7,00	0,13	>0,9999	112,8	22,32	0,999	123,0	19,54	0,999	1,07	5	-7,25	0,11	1,000	87,8	27,57	0,999	114,4	20,12	0,999	0,4	5
**R60T**	-10,02	0,33	1,000	110,7	10,59	0,999	100,7	12	>0,999	1,24	3	-7,16	0,08	1,000	88,5	15,76	0,999	106,1	13,56	1,000	0,9	5	-7,21	0,16	1,000	81,9	10,82	0,999	99,1	10,16	>0,9999	0,4	5
**D63N**	-8,61	0,08	0,002	36,4	6,91	0,0002	21,1	4	0,034	219,2	3	ND	ND	ND	10,2	1,12	<0,0001	24,1	6,26	0,111	ND	3	ND	ND	ND	7,7	0,15	0,0002	12,6	2,75	0,106	ND	3
**T76M**	-9,95	0,17	1,000	112,2	9,56	0,999	95,9	14	1,000	0,84	3	-6,87	0,21	>0,9999	62,5	15,13	0,103	65,0	11,23	0,945	1,64	5	-7,02	0,16	>0,9999	68,1	13,25	0,770	75,5	15,52	0,999	0,6	5
**P86S**	-8,62	0,12	0,002	76,1	11,41	0,931	39,8	10	0,329	18,56	3	ND	ND	ND	21,4	8,98	<0,0001	24,3	10,00	0,010	ND	5	ND	ND	ND	22,2	2,91	<0,0001	19,1	4,92	0,023	ND	5
**H88N**	-9,61	0,20	0,986	95,8	14,55	1,000	78,5	10	0,999	2,44	3	ND	ND	ND	47,4	2,06	0,002	66,1	10,11	0,962	ND	5	-7,14	0,13	1,000	57,2	8,90	0,220	74,0	12,65	0,999	1,3	5
**V96E**	ND	ND	ND	11,2	1,92	<0,0001	19,7	8	0,028	#DIV/0!	3	ND	ND	ND	19,5	5,11	<0,0001	34,5	9,88	0,053	ND	5	ND	ND	ND	10,5	2,69	<0,0001	27,0	8,92	0,070	ND	5
**D103N**	-10,37	0,15	1,000	85,2	6,56	0,999	97,8	9	>0,9999	0,76	3	-7,07	0,15	1,000	81,3	16,57	0,986	94,4	18,14	1,000	1,27	5	-6,95	0,15	>0,9999	96,7	10,14	1,000	99,1	12,22	>0,9999	1,5	5
**R111Q**	-10,00	0,38	1,000	107,1	15,08	1,000	94,7	16	1,000	1,14	3	-6,84	0,19	1,000	64,2	20,40	0,151	71,2	20,04	0,993	4,01	5	-6,90	0,12	1,000	112,2	20,96	0,999	106,3	22,21	1,000	1,6	5
**P114A**	-10,63	0,05	0,999	103,6	12,54	1,000	120,4	11	0,999	0,69	3	ND	ND	ND	49,7	13,42	0,003	44,8	12,87	0,223	ND	5	-7,01	0,09	>0,9999	89,0	21,79	0,999	96,0	20,83	1,000	1,3	5
**R116H**	-9,26	0,25	0,209	69,6	14,66	0,325	49,6	15	0,424	9,43	4	-6,49	0,29	0,999	94,7	12,08	1,000	84,1	19,31	0,999	2,15	4	-6,36	0,15	0,999	121,8	9,84	0,999	96,9	11,21	>0,9999	4,4	4
**M123I**	-10,35	0,26	1,000	88,6	7,97	0,999	95,6	14	1,000	0,8	4	-6,95	0,29	>0,9999	110,0	22,68	0,999	115,9	22,08	0,999	0,7	4	-7,00	0,17	>0,9999	141,3	17,66	0,463	144,7	16,21	0,938	1,1	4
**G125C**	-9,84	0,12	0,999	58,4	23,66	0,022	50,8	15	0,473	3,2	4	-6,96	0,10	>0,9999	78,3	13,71	0,982	82,5	11,40	0,999	0,75	4	-6,97	0,13	>0,9999	92,4	7,00	1,000	94,8	9,95	1,000	1,1	4
**I128M**	-10,38	0,05	1,000	78,0	26,42	0,973	86,1	20	1,000	1,25	3	-6,69	0,28	1,000	78,2	10,67	0,981	75,3	15,02	0,999	1,48	4	-6,99	0,14	>0,9999	144,7	32,27	0,310	143,9	22,19	0,949	1,3	4
**A135T**	-10,54	0,07	0,999	90,2	0,30	0,999	102,5	2	>0,9999	2,36	3	-6,57	0,24	1,000	89,7	13,87	0,999	77,1	9,58	0,999	2,23	3	-6,62	0,04	0,999	101,2	14,02	>0,9999	82,5	8,77	1,000	2,1	3
**A159T**	-9,82	0,19	0,999	94,6	10,78	1,000	86,0	15	1,000	6,91	3	ND	ND	ND	5,2	5,09	<0,0001	30,0	9,29	0,072	ND	4	ND	ND	ND	6,4	3,78	<0,0001	24,2	7,19	0,118	ND	4
**I206T**	-10,50	0,09	0,999	87,3	1,61	0,999	99,7	3	>0,9999	2,56	3	-6,51	0,06	1,000	79,7	16,88	0,994	64,6	9,77	0,993	2,73	3	-6,49	0,11	1,00	84,5	5,19	0,999	64,0	5,10	0,999	3,1	3
**D208N**	-10,72	0,13	0,993	68,5	10,43	0,516	88,3	9	1,000	1,56	3	-6,92	0,10	>0,9999	63,5	10,23	0,508	61,4	8,57	0,985	1,02	3	-6,96	0,17	>0,9999	78,9	6,45	0,999	76,0	7,77	0,999	1,2	3
**D209N**	-9,76	0,26	0,999	93,2	16,37	1,000	81,0	18	0,999	2,98	3	-6,96	0,13	>0,9999	93,3	11,29	1,000	87,2	7,10	1,000	2,11	3	-7,06	0,10	>0,9999	98,3	4,80	>0,9999	101,2	4,77	>0,9999	2,3	3

**A220V**	-9,98	0,37	1,000	131,8	24,88	0,495	132,4	22	0,998	2,3	3	-7,12	0,07	1,000	95,4	10,16	1,000	93,7	6,64	1,000	1,52	3	-7,16	0,07	1,000	98,9	2,22	>0,9999	107,6	3,61	1,000	1,9	3
**V221L**	-10,30	0,15	1,000	95,8	11,56	1,000	99,1	9	>0,9999	2,49	3	-7,24	0,12	1,000	93,5	6,34	1,000	95,2	6,61	1,000	1,1	3	-7,49	0,10	0,999	93,6	14,34	1,000	121,1	8,99	0,999	0,9	3
**R225H**	-8,56	0,41	<0,0001	69,8	22,09	0,181	49,9	22	0,250	86,44	5	-7,17	0,22	1,000	18,6	1,64	<0,0001	20,9	3,22	0,077	1,88	3	-7,04	0,12	>0,9999	32,8	1,68	0,030	36,6	2,66	0,647	6,2	3
**V236M**	-10,77	0,03	0,983	56,3	14,20	0,054	77,8	11	0,999	1,39	3	-6,68	0,11	1,000	59,2	7,82	0,290	48,3	8,87	0,768	1,64	3	-6,51	0,12	0,999	79,9	8,93	0,999	62,5	5,43	0,994	3,1	3
**Y239C**	-10,51	0,09	0,999	84,6	2,88	0,999	98,5	4	>0,9999	2,48	3	-5,91	0,10	0,994	49,8	6,69	0,056	31,6	5,25	0,242	3,18	3	-6,42	0,04	0,999	67,6	6,45	0,969	51,7	4,10	0,966	3,9	3
**D299N**	-9,72	0,12	0,999	106,9	8,99	1,000	89,6	11	1,000	1,43	3	-7,28	0,14	1,000	100,3	9,68	>0,9999	106,7	9,24	1,000	1,26	3	-7,70	0,03	0,999	131,9	17,26	0,974	169,2	13,89	0,471	1,2	3
**L307P**	-10,77	0,11	0,984	88,2	6,00	0,999	103,2	3	1,000	1,37	3	-6,46	0,20	0,999	98,6	14,33	>0,9999	75,9	12,60	0,999	1,26	3	-6,82	0,05	1,000	96,3	1,10	1,000	82,7	3,71	1,000	1,6	3
**R308W**	-7,39	1,15	<0,0001	42,2	11,64	0,001	16,7	10	0,018	191099	3	ND	ND	ND	9,6	3,92	<0,0001	18,6	5,89	0,058	ND	3	ND	ND	ND	2,1	1,29	<0,0001	3,9	3,00	0,043	ND	3
**V311I**	-9,29	0,19	0,503	106,9	8,33	1,000	71,6	8	0,999	4,72	3	-6,61	0,19	1,000	90,7	12,26	1,000	76,4	10,69	0,999	6,23	3	-7,04	0,01	>0,9999	123,2	12,90	0,999	125,14	10,94	0,999	5,4	3
**E362K**	-9,48	0,14	0,906	67,0	5,81	0,417	48,2	6	0,636	27,55	3	ND	ND	ND	6,9	1,31	<0,0001	6,2	4,99	0,012	ND	3	-5,28	0,31	0,250	14,3	3,58	0,001	5,74	3,18	0,053	85,3	3
**V368M**	ND	ND	ND	5,4	4,53	<0,0001	8,7	6	0,005	#DIV/0!	3	-6,96	0,07	>0,9999	65,0	7,25	0,593	60,8	6,86	0,984	2,41	3	-7,10	0,10	>0,9999	112,8	27,09	0,999	115,70	19,71	1,000	5,4	3
**R378C**	-6,30	1,60	<0,0001	11,3	3,51	<0,0001	10,4	4	0,007	1,9E+07	3	ND	ND	ND	4,1	2,37	<0,0001	5,5	5,17	0,010	ND	3	ND	ND	ND	0,6	0,61	<0,0001	6,15	3,18	0,055	ND	3
**A380T**	-10,03	0,26	1,000	100,6	16,61	>0,9999	101,8	22	>0,9999	1	6	-7,18	0,11	1,000	91,2	13,23	1,000	91,5	8,59	1,000	1,49	3	-7,50	0,16	0,999	126,1	6,75	0,994	156,39	12,59	0,839	2,4	3

All data were fitted with the three-parameter logistic curve to obtain EC50, Emax, and AUC. EC50 represent the negative logarithm of agonist concentration in molar that produces half the maximal response. Fmut signaling is the fold change of EC50 mutant divided by EC50 wildtype from the individual experiment. Emax is characterized as the maximal response at (10 nM for cAMP; 10 µM for β-arrestin) normalized to the glucagon receptor wildtype's response. AUC is the integrated response of these two parameters EC50 and Emax. N.D. no EC50 detected in response to glucagon. Data represent the mean ± s.e.m. of 33 (wildtype) or 3–6 (variants) independent experiments performed in duplicate for cAMP and 31 (wildtype) or 3-5 (variants) for β-arrestin1/2. Statistical significance between wildtype and variants Emax, EC50, and AUC was assessed using an ordinary one-way ANOVA test.

Supplementary Table 3 | Binding characteristics of selected impaired activated missense variants

Missense variant	K_D	p-value	Fmut (K_D mutation/K_D wildtype	B_{max} (% of wildtype)	p-value	n
Wildtype	-7.47 ± 0.08		1.00	100 ±		13
M29^{ECD}T	-6.85 ± 0.07	0.004	5.17	71 ± 14	0.1192	4
G40^{ECD}S	-7.29 ± 0.10	0.9949	1.12	120 ± 3	0.7609	3
D63^{ECD}N	-8.50 ± 0.19	<0.0001	0.16	0.2 ± 0.1	<0.0001	4
P86^{ECD}S	-7.59 ± 0.21	0.999	0.95	5 ± 1.2	<0.0001	5
H88^{ECD}N	-7.87 ± 0.04	0.3306	0.53	17 ± 5	<0.0001	3
V96^{ECD}E	-7.63 ± 0.21	0.9878	1.10	1.6 ± 0.7	<0.0001	5
R116^{ECD}H	-6.82 ± 0.10	0.0019	6.25	11 ± 0.8	<0.0001	4
G125^{ECD}C	-7.70 ± 0.12	0.9639	0.97	1.5 ± 0.3	<0.0001	3
A159$^{1.57}$T	-7.98 ± 0.11	0.0798	0.39	7 ± 0.8	<0.0001	3
D209^{ECL1}N	-7.50 ± 0.18	0.9999	1.39	27 ± 13	<0.0001	3
A220$^{3.25}$V	-7.43 ± 0.02	0.9998	1.50	22 ± 5	<0.0001	3
V221$^{3.26}$L	-7.20 ± 0.07	0.8948	1.39	152 ± 19	0.0006	3
R225$^{3.30}$H	-7.29 ± 0.01	0.9947	1.76	5 ± 0.7	<0.0001	3
V236$^{3.41}$M	-7.81 ± 0.09	0.4822	0.83	63 ± 27	0.0129	4
R308$^{5.40}$W	-7.40 ± 0.13	0.9951	1.51	22 ± 5	<0.0001	4
V311$^{5.43}$I	-7.02 ± 0.12	0.192	1.46	89 ± 16	0.9958	3
E362$^{6.53}$K	-6.48 ± 0.03	<0.0001	12.72	76 ± 13	0.3047	4
V368$^{6.59}$M	-7.25 ± 0.12	0.9381	1.64	30 ± 2	<0.0001	4
R378$^{7.35}$C	-7.76 ± 0.09	0.438	0.71	56 ± 12	0.0016	4

All data were fitted with the three-parameter logistic curve to obtain K_D and B_{max}. K_D represent the negative logarithm of the equilibrium dissociation constant of glucagon for the glucagon receptor wildtype or mutant. Fmut binding is the fold change of KD mutant divided by KD wildtype from the individual experiment. B_{max} is characterized as the maximal specific binding. Data represent the mean ± s.e.m. of 13 (wildtype) or 3–5 (variants) independent experiments performed in duplicate. Statistical significance was assessed using an ordinary one-way ANOVA for K_D and B_{max} (*P < 0.05; **P < 0.01; as compared to wildtype response).

Supplementary Table 4 | Primer sequences for the created missense variants in the glucagon receptor

Missense variant	Forward primer	Reverse primer
P3^{ECD}S	ATGCCCTCCTGCCAGCCACAGCGACCCCTG	CAGGGGTCGCTGTGGCTGGCAGGAGGGCAT
R8^{ECD}Q	GCCCCCCTGCCAGCCACAGCAACCCCTGCTG	CAGCAGGGGTTGCTGTGGCTGGCAGGGGGGC
A26^{ECD}T	CACAGGTCCCCTCCACTCAGGTGATG	CATCACCTGAGTGGAGGGGACCTGTG
M29^{ECD}T	CTCCGCTCAGGTGACGGACTTCCTGTTTGAG	CTCAAACAGGAAGTCCGTCACCTGAGCGGAG
G40^{ECD}S	GTGGAAGCTCTACTCTGACCAGTGTCACCAC	GTGGTGACACTGGTCAGAGTAGAGCTTCCAC
D41^{ECD}E	GCTCTACGGTGAGCAGTGTCACCACAACC	GGTTGTGGTGACACTGCTCACCGTAGAGC
T54^{ECD}M	CTGCCCCCTCCCATGGAGCTGGTGTGCAAC	GTTGCACACCAGCTCCATGGGAGGGGGCAG
R60^{ECD}T	CTCCCACGGAGCTGGTGTGCAACACAACC	GGTTGTGTTGCACACCAGCTCCGTGGGAG
D63^{ECD}N	CCTTCAACAAGTATTCCTGCTGGCCG	CGGCCAGCAGGAATACTTGTTGAAGG
T76^{ECD}M	CACCCCCGCCAATACCATGGCCAACATCTC	GAGATGTTGGCCATGGTATTGGCGGGGGTG
P86^{ECD}S	CCTGCCCCTGGTACCTGTCTTGGCACCAC	GTGGTGCCAAGACAGGTACCAGGGGCAGG
H88^{ECD}N	CTGCCTTGGAACCACAAAGTGCAACACCGC	GCGGTGTTGCACTTTGTGGTTCCAAGGCAG
V96^{ECD}E	GCAACACCGCTTCGAGTTCAAGAGATGCGGG	CCCGCATCTCTTGAACTCGAAGCGGTGTTGC
D103^{ECD}N	GTTCAAGAGATGCGGGCCCAACGGTCAGTG	CACTGACCGTTGGGCCCGCATCTCTTGAAC
R111^{ECD}Q	GACCCCAGGGGCAGCCTTGGCGTGATG	CATCACGCCAAGGCTGCCCCTGGGGTC
P114^{ECD}A	GGACCCCGGGGGCAGGCTTGGCGTGATGCC	GGCATCACGCCAAGCCTGCCCCCGGGGTCC
R116^{ECD}H	CCGGGGGCAGCCTTGGCATGATGCCTCCCAG	CTGGGAGGCATCATGCCAAGGCTGCCCCCGG
M123^{ECD}I	GCCAGATCGATGGCGAGGAGATTGAGGTCC	GGACCTCAATCTCCTCGCCATCGATCTGGC
G125^{ECD}C	GCCTCCCAGTGCCAGATGGATTGCGAGGAG	CTCCTCGCAATCCATCTGGCACTGGGAGGC
I128^{ECD}M	GGCGAGGAGATGGAGGTCCAGAAGGAGGTG	CACCTCCTTCTGGACCTCCATCTCCTCGCC
A135$^{1.33}$T	GAAGGAGGTGACCAAGATGTACAGC	GCTGTACATCTTGGTCACCTCCTTC
A159$^{1.57}$T	CTGCTCCTCACCTTGGCCATCCTGG	CCAGGATGGCCAAGGTGAGGAGCAG
I206^{ECL1}T	CAGAAAACTGGCGACGACCTCAGTG	CACTGAGGTCGTCGCCAGTTTTCTG
D208^{ECL1}N	CCAGAAAATTGGCAACGACCTCAGTG	CACTGAGGTCGTCGCCAATTTTCTGG
D209^{ECL1}N	GAAAATTGGCGACAACCTCAGTGTCAGCAC	GTGCTGACACTGAGGTTGTCGCCAATTTTC
A220$^{3.25}$V	CAGTGATGGAGTGGTGGCTGGCTGC	GCAGCCAGCCACCACTCCATCACTG
V221$^{3.26}$L	CACCTGGCTCAGTGATGGAGCGCTGGCTGG	CCAGCCAGCGCTCCATCACTGAGCCAGGTG
V236$^{3.41}$M	GGCATCATGGCCAACTACTGCTGGC	GCCAGCAGTAGTTGGCCATGATGCC
Y239$^{3.44}$C	GTGGCCAACTGCTGCTGGCTG	CAGCCAGCAGCAGTTGGCCAC
R225$^{3.30}$H	GAGCGGTGGCTGGCTGCCATGTGGCCGCGG	CCGCGGCCACATGGCAGCCAGCCACCGCTC

D299^{ECL2}N	GACCAGCAATAACAACATGGGCTTCTGGTG	CACCAGAAGCCCATGTTGTTATTGCTGGTC
L307$^{5.39}$P	CTTCTGGTGGATCCCGCGGTTCCCC	GGGGAACCGCGGGATCCACCAGAAG
R308$^{5.40}$W	GGTGGATCCTGTGGTTCCCCGTCTTCC	GGAAGACGGGGAACCACAGGATCCACC
V311$^{5.43}$I	CGGTTCCCCATCTTCCTGGCCATCCTGATC	GATCAGGATGGCCAGGAAGATGGGGAACCG
E362$^{6.53}$K	CTCTGCTGGGCGTCCACAAAGTGGTC	GACCACTTTGTGGACGCCCAGCAGAG
V368$^{6.59}$M	GTGGTCTTCGCCTTCATGACGGACGAGCAC	GTGCTCGTCCGTCATGAAGGCGAAGACCAC
R378$^{7.35}$C	CACCCTGTGCTCCGCCAAGCTCTTC	GAAGAGCTTGGCGGAGCACAGGGTG
A380$^{7.37}$T	CTGCGCTCCACCAAGCTCTTCTTCGAC	GTCGAAGAAGAGCTTGGTGGAGCGCAG

Supplementary Figure 1 | dose-dependent DNA activation of V368$^{6.59}$M

Dose-dependent increase (5–20 µg) of transfected wildtype amd V368$^{6.59}$M glucagon receptor DNA resulted in weak activation after stimulation with 10 nM glucagon (n=4). Statistical significance was assessed using a two-way ANOVA (**P < 0.01; ****P < 0.0001 as compared to wildtype response).

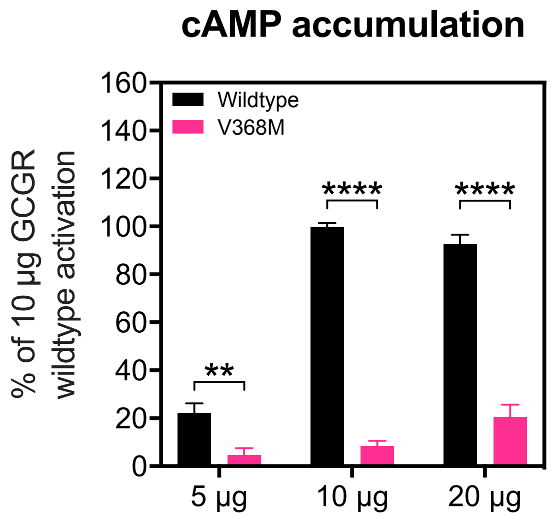
cAMP accumulation
% of 10 µg GCGR wildtype activation
Wildtype
V368M
**
****
****
0
20
40
60
80
100
120
140
160
5 µg
10 µg
20 µg
